# An exploratory analysis on diastolic function in the intensive compared with less intensive blood pressure control to prevent adverse cardiac remodelling in children with chronic kidney disease (HOT-KID): a parallel-group, open-label, multicentre, randomised, controlled trial

**DOI:** 10.1016/j.ebiom.2025.105691

**Published:** 2025-04-21

**Authors:** Haotian Gu, John M. Simpson, Janette Cansick, Eric Finlay, Rodney Gilbert, Andrew Lunn, Heather Maxwell, Henry Morgan, Mohan Shenoy, Rukshana Shroff, Pushpa Subramaniam, Jane Tizard, Yincent Tse, Phil Chowienczyk, Manish D. Sinha, HOT-KID study, Janette Cansick, Abdel Douiri, Eric Finlay, Rodney Gilbert, Haotian Gu, Larissa Kerecuk, Andrew Lunn, Heather Maxwell, Henry Morgan, Reza Razavi, Mohan Shenoy, Rukshana Shroff, Pushpa Subramaniam, Jane Tizard, Yincent Tse, Poothirikovil Venugopalan, John Simpson, Phil Chowienczyk, Manish Sinha

**Affiliations:** a King's College London British Heart Foundation Centre, London, UK; b Department of Paediatric Cardiology, Evelina London Children's Hospital, Guy's & St Thomas NHS Foundation Trust, Westminster Bridge Road, London SE1 7EH, UK; c Department of Paediatrics, Medway Maritime Hospital, Medway, UK; d Department of Paediatric Nephrology, Leeds General Infirmary, Leeds, UK; e Department of Paediatric Nephrology, Southampton General Hospital, Southampton, UK; f Department of Paediatric Nephrology, Nottingham University Hospital NHS Trust, Nottingham, UK; g Department of Paediatric Nephrology, Royal Hospital for Sick Children, Glasgow, UK; h Department of Paediatric Nephrology, Alder Hey Children's Hospital, Liverpool, UK; i Department of Paediatric Nephrology, Royal Manchester Children's Hospital, Manchester, UK; j Department of Paediatric Nephrology, UCL Great Ormond Street Hospital and Institute of Child Health, London, UK; k Department of Paediatrics, St Georges Hospital, Tooting, London, UK; l Department of Paediatric Nephrology, Bristol Royal Hospital for Children, Bristol, UK; m Department of Paediatric Nephrology, Great North Children's Hospital, Newcastle Upon Tyne, UK; n Department of Paediatric Nephrology, Evelina London Children's Hospital, Guy's & St Thomas NHS Foundation Trust, Westminster Bridge Road, London SE1 7EH, UK

**Keywords:** Diastolic function, Blood pressure, Hypertension, Children, Chronic kidney disease

## Abstract

**Background:**

Relationship between blood pressure (BP) control and left ventricular (LV) diastolic function in children with chronic kidney disease (CKD) is uncertain. The aim of this study is to investigate whether achieving lower BP yields a favourable impact on diastolic function.

**Methods:**

We performed an exploratory analysis in the HOT-KID, a parallel group, open-label, multicentre, randomised, controlled trial (ISRCTN25006406). Children with CKD were randomised to standard (50th–75th percentile) or intensive (<40th percentile) standardised office systolic BP targets. Echocardiograms were performed at baseline and at follow-up visits. Diastolic function was assessed by early (E) and late mitral inflow (A) E/A ratio, mitral annular motion of myocardial relaxation (e’) and atrial contraction (a’) velocity, LV compliance of E/e’ and e’/a’ ratio, and left atrial volume index (LAVi) by a blinded observer.

**Findings:**

There was a difference in the average annual rate of change in E/A ratio (difference in means −0·07 per year, 95% CI: −0·14 to −0·01), septal e’ (difference in means −0·003 m/s per year, 95% CI: −0·005 to 0·001), and LAVi (difference in means 0·82 ml/m^2^ per year, 95% CI: 0·22–1·42) in the standard (n = 60) compared to the intensive treatment arm (n = 64). However, the average annual changes in all other diastolic function measures were similar between standard and intensive treatment groups. There was no difference for overall adverse events or serious adverse events between the two treatment groups.

**Interpretation:**

Our exploratory analysis in a small, open label RCT suggests that achieving lower blood pressure may favourably impact some measures of LV diastolic function in children with CKD.

**Funding:**

10.13039/501100000274British Heart Foundation (PG/11/90/28,994); The authors MDS, PJC acknowledge financial support from the Department of Health via the 10.13039/501100000272National Institute for Health Research (NIHR) comprehensive Biomedical Research Centre and Clinical Research Facilities awards to Guy’s and St Thomas’ NHS Foundation Trust in partnership with King’s College London and 10.13039/100010872King's College Hospital NHS Foundation Trust. There are no relationships with industry.

**Research in context**

**Evidence before this study**

In children with chronic kidney disease (CKD), there exists a complex interplay between left ventricular (LV) remodelling, diastolic function, and elevated blood pressure (BP). Structural adaptations, such as concentric hypertrophy in response to increased afterload can impair diastolic function by reducing myocardial compliance. Besides BP, additional factors including anaemia, male sex, and hypoalbuminemia are implicated in alterations of cardiac structure and function. The Hypertension Optimal Treatment in Children with Chronic Kidney Disease (HOT-KID) trial demonstrated that optimal BP control, with a target systolic BP near the 50th percentile, is associated with a reduction in relative wall thickness (RWT). These findings build on the renoprotective outcomes observed in the Progression of CKD in Paediatric Patients (ESCAPE) trial, which underscored the benefits of lowering ambulatory BP targets to decelerate CKD progression. Given that diastolic dysfunction serves as an early marker of preclinical heart failure - particularly in adult CKD populations where it is linked to adverse outcomes – the association between BP and LV diastolic function in children warrants close examination.

**Added value of this study**

This study examines the effect of different levels of BP control on left ventricular diastolic function in children with CKD using data from the HOT-KID randomised controlled trial. Our findings suggests that more intensive as compared with standard blood pressure control has a favourable impact on some measures of diastolic function including the E/A ratio, septal e’ and LAVi in children with CKD. This finding suggests that lower BP may play an important role in preserving diastolic function in children with CKD.

**Implications of all the available evidence**

Although our exploratory analyses are subject to limited power, they support the concept that targeting lower BP levels may prevent the progression of adverse diastolic function progression in children with CKD. If our findings are confirmed by further studies and combined with results of the primary outcome measures from the HOT-KID trial and the ESCAPE study, a BP threshold at the 50th percentile may be recommended as likely to prevent both adverse cardiac remodelling and diastolic dysfunction.

## Introduction

In children with chronic kidney disease (CKD), there is an intricate relationship between adverse left ventricular (LV) remodelling, systolic and diastolic function and elevated blood pressure (BP).^1^, ^2^, ^3^, ^4^, ^5^, ^6^ Moreover, structural changes such as concentric hypertrophy, where the left ventricle thickens to compensate for increased afterload, may contribute to diastolic dysfunction by altering the compliance of the myocardium.^7^ Increased afterload prompts adaptive changes in the myocardium, influencing both diastolic function and left ventricular mass (LVM). In addition to BP, several other factors including anaemia, male sex and low albumin have been shown to impact on cardiac structure and function. We conducted a search on PubMed, Embase, and CENTRAL using the keywords “diastolic function”, “children”, “chronic kidney disease”, “hypertension” and “randomized controlled trial.” and found no clinical trial exploring the impact of lowering BP on diastolic function in children with CKD.

We recently reported results of the open-label randomised controlled trial, the Hypertension Optimal Treatment in Children with Chronic Kidney Disease (HOT-KID) study and showed that cardiac remodelling as measured by LV relative wall thickness (RWT) in children with CKD is related to BP control with an optimal office systolic BP target near the 50th percentile for preventing increased LVM.^8^ Our data added to the renoprotective findings of the Progression of CKD in Paediatric Patients (ESCAPE) trial, that showed the benefit of lowering ambulatory BP targets on slowing kidney disease progression.^9^

Diastolic dysfunction is an early indicator of pre-clinical heart failure and is particularly significant in adults with CKD, where it is linked to a worse prognosis.^10^^,^^11^ Previous research has highlighted an association between elevated BP and diastolic dysfunction in children with CKD.^1^ Thus, understanding the intricate interplay between BP and LV diastolic function in this population is pivotal for highlighting the relevance of BP targets and for understanding the progression of cardiovascular complications in childhood CKD. We therefore performed an exploratory study to investigate whether achieving lower BP yields a favourable impact on diastolic function in the HOT-KID study.

## Methods

### Study design and participants

The HOT-KID trial was a parallel-group, open-label, multicentre, randomised, controlled trial (ISRCTN number: ISRCTN25006406) conducted at 14 clinical centres across England and Scotland (Table 1). The full trial protocol was previous published.^8^

**Table 1:** List of participating centres in the UK.

Institution	City	Country
Evelina London Children's Hospital	London	England, UK
Medway Maritime Hospital	Medway	England, UK
Leeds General Infirmary	Leeds	England, UK
Southampton General Hospital	Southampton	England, UK
Birmingham Children's Hospital	Birmingham	England, UK
Nottingham University Hospital	Nottingham	England, UK
Royal Hospital for Sick Children	Glasgow	Scotland, UK
Alder Hey Children's Hospital	Liverpool	England, UK
Royal Manchester Children's Hospital	Manchester	England, UK
Great Ormond Street Hospital	London	England, UK
St George's Hospital	London	England, UK
Bristol Royal Hospital for Children	Bristol	England, UK
Great North Children's Hospital	Newcastle Upon Tyne	England, UK
Royal Alexandra Children's Hospital	Brighton	England, UK

Children aged 2–15 years with stages 1–4 CKD (eGFR >15 ml/min/1·73 m^2^) were recruited to the study. Detailed inclusion and exclusion criteria, along with the trial protocol, have been previously published.^8^ In brief, patients were randomised according to their standardised office systolic BP with BP targeted to either standard (50th −75th percentile, Z score 0·00–0·67) or intensive (<40th percentile, Z score −0·26) treatment groups.

### Ethics

The trial design complies with the principles of the Declaration of Helsinki and was approved by the Clinical Studies Group of the British Association for Paediatric Nephrology and UK National Research Ethics Committee (10/H0802/13), participating institutions, and relevant regulatory authorities. Parents or legal guardians of children gave written informed consent, and participants gave assent as appropriate.

### Randomisation and masking

Randomisation and masking processes have been previously published.^8^ In brief, participants were randomly assigned (1:1) to receive standard treatment or intensive treatment by the chief investigator (MDS) using a web-based randomisation system. Participants and clinicians were informed of allocation of the participants, but were masked to systolic BP percentile and target and results.

### Procedures

Following randomisation, antihypertensive treatment was initiated or up-titrated in children not meeting BP targets. Adjustment of antihypertensive drugs during the trial followed previously described protocols.^8^ Trial-related BP targets for all participants were provided at baseline and at annual intervals throughout their participation.^12^

#### BP monitoring

Measurement of clinic BP was performed by the same trained staff member according to a standardised protocol.^8^ In brief, office BP was measured by a standardised method, three times using auscultation and aneroid sphygmomanometer with an appropriately size cuff and the average of three measurements calculated.

#### Data handling procedures

Data were collected on standardised data collecting forms and entered into an electronic database (MedSciNet). Data checking, completion and validation were performed by the trial statistics unit.

#### Echocardiography

Transthoracic echocardiograms were conducted at baseline and annual intervals by a core echo team from the lead centre (Evelina London Children's Hospital; King's College London, UK), following a standardised research protocol and according to the American Society of Echocardiography guidelines.^13^ The final image analysis was carried out by a single investigator who was blinded to the treatment arm allocation.

LV diastolic function was assessed according to the American Society of Echocardiography guidelines.^13^ Mitral inflow E wave (mitral valve blood flow of early passive diastolic filling) velocity and A wave (mitral valve blood flow of atrial contraction) velocity were measured using Pulsed wave Doppler at the tip of mitral valve leaflets from an apical 4-chamber view. Mitral annulus diastolic motion (e’ and a’) were recorded using Tissue Doppler imaging (TDi) at the basal mitral annulus (lateral and septal). Average e’ was calculated as (lateral e’ + septal e’)/2. Left atrial volume (LAV) was measured at end-systole from apical 4-chamber and 2-chamber views with indexation to body surface area (LAVi).^14^ E/A ratio, E/lateral e’, E/septal e’, E/average e’, lateral e’/a’, septal e’/a’ and average e’/a’ were calculated as markers of LV diastolic function.^13^ Abnormal diastolic function was defined as E/A <1·0, average e’/a’ <1·0 and E/average e’ >8·0 (with E/average e’ > 14·0 considered to be clinically significant).^1^ Normal reference range of Doppler mitral inflow (E wave and A wave) for children were from a previously published study.^15^ LVM was indexed (LVMi) to height powered 2·7 and RWT was calculated using a previously described formula.^8^

### Outcomes

Primary and secondary outcomes have been previously reported.^8^ In brief, in the original HOT-KID trial, these were the difference in LVMi and RWT between the two treatment groups. Since the current study is an exploratory analysis on diastolic function, there were no pre-defined outcome measures.

### Sample size

The original sample size calculation was set to detect a difference in change in mean LVMi as previously reported.^8^ The sample size for this exploratory analysis was based on feasibility.

### Statistics

All results are expressed as mean ± standard deviation (SD). Non-normally distributed measurements were log-transformed before statistical testing.

LV diastolic function measures were compared between the two trial arms based on an intention-to-treat basis, defined as all the children who underwent randomisation irrespective of the BP target achieved. There was no allowance for multiplicity. The mean annual rate of change and the difference in these rates were estimated using a mixed effects model using a restricted maximum likelihood-based repeated measures approach in combination with the Newton Raphson Algorithm. Analyses included the fixed, categorical effects of treatment, clinical centre, visit, and treatment-by-visit interaction, as well as the continuous, fixed covariates of baseline value and baseline score-by-visit interaction. A common unstructured variance structure was used to model the within-patient errors. The Kenward-Roger approximation was used to estimate denominator degrees of freedom. This model considered baseline and all subsequent follow-up measurements, accommodating participants with missing measurements. This analysis was repeated in subjects who completed 3 years of follow up. Linearity was assessed by Scatter Plots with residuals and predicted values. Patient follow-up ranged from 1 to 5 years, and, like all linear mixed models, it assumed that missing data were missing-at-random. A further sensitivity analysis was performed to evaluate the possible influence of missing values for the key outcomes of diastolic function reported. Multiple Imputation was done using Markov chain Monte Carlo (MCMC) with the following variables used to impute the missing values: centre, group, time and baseline values. Twenty datasets were imputed to generate the average adjusted mean difference.

Additional linear regression analysis was performed to assess the relationship between the annual rate of change in diastolic function measures and LV structures. Statistical analysis was performed using SPSS 29·0 (SPSS Inc, Chicago, IL), and a two-sided p-value less than 0·05 was considered significant.

### Role of funders

The funder of the study was not involved in study design, data collection, data analysis, data interpretation, or writing of the report, other than providing external review at the stage of funding application.

## Results

### Study population

A total of 124 participants were randomised with 64 in the intensive arm and 60 in the standard arm. Detailed study flow chart is shown in Figure S1. The participants were followed for a median duration of 38·7 months (IQR 28·1, 52·2), and the final study visit occurred on March 5, 2019 as previously reported.^8^

### Baseline characteristics

Baseline characteristics were well balanced between the two trial groups as previously reported (Table 2).^8^ In brief, the mean age of all participants was 10·0 ± 3·5 years, 69 (55·6%) of 124 participants were male (sex was self-reported by study participants), and 107 (86%) were of white ethnicity. Mean clinic BP was 107/63 mmHg (SD 11/12), with a mean BP Z score of 0·55/0·06 (0·91/1·25).^8^

**Table 2:** Baseline characteristics of the intention-to-treat population (General Demographics and Biochemistry Data were previously reported in [8]).

	Intensive arm (n = 64)	Standard arm (n = 60)
Age (years)	9·4 (6·2–12·4)	10·3 (7·3–12·8)
Sex n (%)		
Male	34 (53)	35 (58)
Female	30 (47)	25 (42)
Ethnicity [n (%)]		
Asian	6 (9)	6 (10)
Black	0 (0)	2 (3)
White	57 (89)	50 (83)
Other	1 (2)	2 (3)
Primary Renal Disease [n (%)]		
CAKUT	39 (61)	42 (70)
Glomerulopathies	5 (8)	7 (12)
Other	20 (31)	11 (18)
Height (cm)	134·0 (114·7–151·5)	137·7 (124·6–150·5)
Weight (kg)	30·9 (21·1–42·4)	32·9 (25·4–94·3)
BMI (kg/m^2^)	17·0 (15·9–19·1)	18·0 (16·0–22·0)
Heart rate (bpm)	81 ± 13·1	82 ± 14·5
SBP (mmHg)	107 ± 11	108 ± 11
DBP (mmHg)	62 ± 11	64 ± 13
SBP z-score	0·54 ± 0·98	0·57 ± 0·84
DBP z-score	0·02 ± 1·23	0·10 ± 1·29
MAP (mmHg)	77 ± 10	78 ± 11
Antihypertensive Medications n (%)	43 (67·2)	37 (61·7)
Biochemistry		
eGFR (ml/min/1·73 m^2^)	73·6 (57·3–94·4)	83·5 (59·6–98·8)
Haemoglobin (g/L)	124·0 (111·0–130·0)	130·0 (121·0–140·0)
Albumin (g/L)	46·0 (42·0–47·0)	43·0 (41·0–48·0)
Serum corrected Calcium (mmol/L)	2·47 (2·37–2·56)	2·39 (2·33–2·44)
Serum phosphate (mmol/L)	1·40 (1·23–1·60)	1·40 (1·30–1·54)
Serum iPTH (ng/L)	27·0 (6·65–63·0)	29·0 (9·68–40·5)
25 (OH) vitamin D3 (microg/L)	60·0 (44·0–88·0)	65·2 (41·0–86·0)
Diastolic function measurements		
E wave (m/s)	0·99 ± 0·15	0·98 ± 0·15
A wave (m/s)	0·52 ± 0·15	0·53 ± 0·15
E/A ratio	2·0 ± 0·6	2·0 ± 0·6
E/A < 1·0 n (%)	0	0
Lateral e’ wave (m/s)	0·17 ± 0·04	0·16 ± 0·03
Lateral a’ wave (m/s)	0·066 ± 0·02	0·064 ± 0·02
Lateral e’/a’ ratio	2·7 ± 1·0	2·7 ± 0·8
Septal e’ wave (m/s)	0·13 ± 0·02	0·12 ± 0·02
Septal a’ wave (m/s)	0·06 ± 0·02	0·06 ± 0·01
Septal e’/a’ ratio	2·2 ± 0·7	2·3 ± 0·7
E/Lateral e’ ratio	6·1 ± 1·5	6·3 ± 1·5
E/Septal e’ ratio	8·1 ± 1·8	8·0 ± 1·5
E/Ave e’ ratio	6·9 ± 1·4	7·0 ± 1·4
E/Ave e’ ratio >8·0 n (%)	16 (25·0%)	11 (18·3%)
Ave e’/a’ ratio	2·4 ± 0·6	2·5 ± 0·6
Ave e’/a’ ratio <1·0 n (%)	0	0
Left atrial volume (ml)	24·6 ± 10·9	23·6 ± 11·5
Left atrial volume index (ml/m^2^)	21·9 ± 5·5	19·4 ± 5·6

CAKUT, congenital anomaly of the kidney and urinary tract; SDS, standard deviation score; SBP, systolic blood pressure; DBP, diastolic blood pressure; MAP, mean arterial pressure; eGFR, estimated glomerular filtration rate; UAlb/Creat, urinary albumin creatinine ratio; iPTH, intact parathyroid hormone; E wave: mitral inflow early diastolic filling; A wave: mitral inflow atrial contraction filling; Lateral e’: mitral lateral early diastolic annular motion; lateral a’: mitral lateral annular diastolic atrial contraction motion; septal e’: mitral septal early diastolic annular motion; septal a’: mitral septal annular diastolic atrial contraction motion.

### Echocardiography

#### Baseline measures

Participants were matched for most LV geometry variables as previously described^8^ and diastolic function measures at baseline (Table 2). There was no participant with E/A or average e’/a’ ratios <1·0; E/average e’ >8·0 was found in 27 (21·8%) of 124 children at baseline [16 (25%) of 64 participants in the intensive treatment group vs 11 (18·3%) of 60 in the standard treatment group]. No participant had clinically significant diastolic dysfunction as measured by E/average e’ greater than 14. Children in the intensive arm had larger LAVi (21·9 ± 5·5 ml/m^2^) compared to those in the standard arm (19·4 ± 5·6 ml/m^2^).

#### Change in mitral inflow measures

**Fig. 1:**
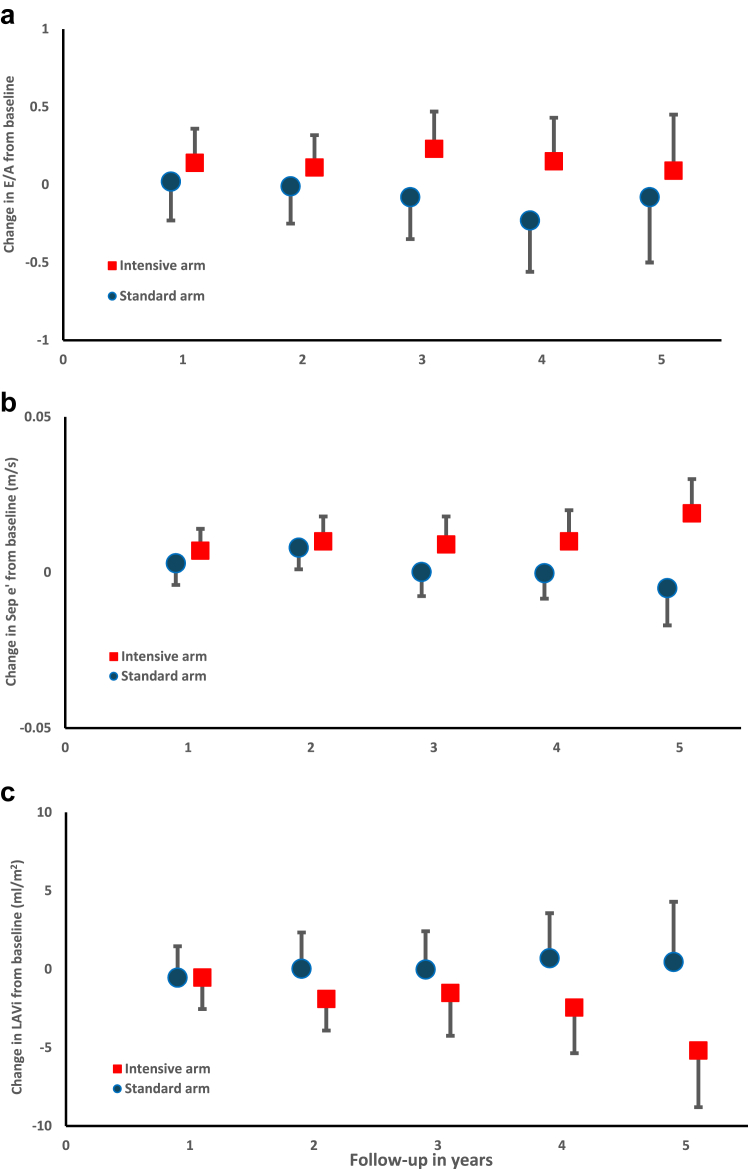
Diastolic function measures over time (a) Change in mean E/A ratio from baseline. (b) Change in mean Septal e’ velocity from baseline. (c) Change in mean left atrial volume index (LAVi) from baseline. Means were estimated by use of a linear mixed effects model for repeated measures. Data are presented as means with 95% confidence intervals. At the final follow up visit the majority of patients had not reached 5 years of study participation, which accounts for the sharp decrease in numbers available for follow-up between year 4 and 5.

There was a statistically significant difference in the annual rate of change in the E/A ratio. (−0·07 per year, 95% CI: −0·14 to −0·01 per year) between standard and intensive treatment arms (Table 3a and Fig. 1a). At 3 years the estimated difference in the mean E/A ratio was −0·37, 95% CI: −0·64 to −0·11 (Table 3a), but there was no statistically significant difference in the annual rate of change in E wave (difference in mean −0·001 m/s, 95% CI: −0·01, 0·01) or A wave (difference in mean −0·01 m/s, 95% CI: −0·02, 0·02) between standard and intensive arms. No participants had E/A ratio <1 throughout the trial.

**Table 3:** Diastolic function measures over time of the intention-to-treat population.

a: Change in mean E/A ratio from baseline			
E/A ratio	Intensive arm (SD)	Standard arm (SD)	Difference (95% CI)
Change in per year, mean	0·04 (0·16)	−0·04 (0·51)	−0·07 (−0·14, −0·01)
Baseline (n = 64, 60)	2·03 (0·67)	1·97 (0·57)	−0·06 (−0·28, 0·16)
Y1 (n = 51, 54)	2·17 (0·73)	1·99 (0·60)	−0·18 (−0·43, 0·07)
Y2 (n = 47, 47)	2·13 (0·65)	1·95 (0·52)	−0·18 (−0·41, 0·05)
Y3 (n = 36, 32)	2·26 (0·61)	1·89 (0·52)	−0·37 (−0·64, −0·11)
Y4 (n = 23, 22)	2·18 (0·74)	1·74 (0·53)	−0·44 (−0·81, −0·07)
Y5 (n = 11, 11)	2·11 (0·70)	1·88 (0·47)	−0·23 (−0·69, 0·23)

b: Change in mean Septal e’ (m/s) from baseline			
Septal e’ in m/s	Intensive arm (SD)	Standard arm (SD)	Difference (95% CI)
Change in per year, mean	0·003 (0·01)	0·0001 (0·004)	−0·003 (−0·005, −0·001)
Baseline (n = 64, 60)	0·125 (0·02)	0·124 (0·02)	−0·001 (−0·008, 0·006)
Y1 (n = 51, 54)	0·132 (0·03)	0·127 (0·01)	−0·005 (−0·01, 0·003)
Y2 (n = 47, 47)	0·136 (0·01)	0·132 (0·02)	−0·004 (−0·01, 0·005)
Y3 (n = 36, 32)	0·134 (0·02)	0·124 (0·02)	−0·01 (−0·02, −0·0004)
Y4 (n = 23, 22)	0·136 (0·03)	0·124 (0·01)	−0·01 (−0·02, −0·002)
Y5 (n = 11, 11)	0·145 (0·03)	0·119 (0·04)	−0·03 (−0·05, −0·002)

c: Change in left atrial volume index (ml/m^2^) from baseline			
Left atrial volume index (ml/m^2^)	Intensive arm (SD)	Standard arm (SD)	Difference (95% CI)
Change per year, mean	−0·73 (3·42)	0·09 (4·16)	0·82 (0·22, 1·42)
Baseline (n = 64, 60)	21·9 (5·71)	19·4 (5·93)	−2·45 (−4·43, −0·47)
Y1 (n = 51, 54)	21·3 (5·83)	18·9 (5·25)	−2·40 (−4·52, −0·26)
Y2 (n = 47, 47)	20·0 (5·24)	19·5 (6·64)	−0·49 (−2·88, 1·89)
Y3 (n = 36, 32)	20·2 (7·65)	19·4 (6·05)	−0·74 (−4·0, 2·52)
Y4 (n = 23, 22)	19·4 (5·19)	20·2 (5·93)	0·73 (−3·30, 4·77)
Y5 (n = 11, 11)	16·7 (3·96)	19·9 (5·80)	3·19 (−2·68, 9·07)

Means were estimated by use of a linear mixed effects model for repeated measures. At the final follow up visit the majority of patients had not reached 5 years of study participation, which accounts for the sharp decrease in numbers available for follow-up between year 4 and 5.

#### Change in Tissue Doppler measures

Intensive BP control resulted in an increase in Septal e’ over time (Fig. 1b). The annual rate of change in Septal e’ was greater in the intensive arm than in the standard arm: difference in means −0·003 m/s (95% CI −0·005 to 0·001) per year (Table 3b). There was no statistically significant difference in the annual rate of change in septal a’ (difference in means 0·0 m/s, 95% CI: −0·001, 0·001), lateral e’ (difference in means 0·004 m/s, 95% CI: −0·003, 0·01) or lateral a’ (difference in means 0·001 m/s, 95% CI: −0·001, 0·002) between standard and intensive groups.

There was no statistically significant difference in the annual rate of change in E/lateral e’ (difference in means −0·04, 95% CI: −0·167, 0·0086), E/septal e’ (difference in means 0·167, 95% CI: −0·02, 0·035), E/average e’ (difference in means −0·104, 95% CI: −0·22, 0·012), lateral e’/a’ (difference in means −0·026, 95% CI: −0·096, 0·044), septal e’/a’ or average e’/a’ (difference in means 0·28, 95% CI: −0·23, 0·80) between standard and intensive groups. Five (13·9%) of 36 in the intensive treatment group and 6 (18·8%) of 32 in the standard treatment group had E/average e’ greater than 8 at 3 years, p = 0·59. Over the course of the trial, only one participant in the standard treatment group had clinically significant diastolic dysfunction (E/average e’ = 16·2) at year 5.

#### Change in left atrial volume index

Intensive BP treatment resulted in a reduction in LAVi over time (Fig. 1c). The annual rate of change in LAVi was greater in the intensive arm than in the standard arm: difference in means 0·82 ml/m^2^ (95% CI 0·22–1·42) per year (Table 3c).

Additional linear mixed model analysis adjusted for baseline eGFR (categorised as 60 ml/min per 1·73 m^2^ and median value of 77 ml/min per 1·73 m^2^) showed unchanged results for E/A, septal e’, and LAVi. We observed similar findings when the analyses were repeated in subjects who completed 3-years of follow-up (Table S1).

There were 27 missing interim visits out of 485 planned visits between randomisation and final visit (4 in Y1, 10 in Y2, 9 in Y3, and 4 in Y4). Results using multiple imputation analysis to examine the potential influence of missing data produced similar results. In particular, differences in E/A ratio, Septal e’ and LAVi were similar to those in the original data (difference in means: E/A −0·70, 95% CI −0·14 to −0·001; Septal e’ −0·003 m/s, 95% CI −0·005 to −0·001; LAVi: 0·81 ml/m^2^, 95% CI: 0·21–1·42).

There was no statistically significant relationship between the annual rate of change in diastolic function measures (E/A ratio and septal e’) and LV structure measures (LVMi and RWT). However, the annual rate of change in LAVi was positively associated with the annual rate of change in LVMi and RWT (Table S2).

#### Safety

As previously reported, there was no difference in risk of adverse events between the two treatment groups: the risk difference 0·02 (95% CI −0·16 to 0·19, p = 0·83) for overall adverse events and 0·07 (−0·05 to 0·19, p = 0·25) for serious adverse events.^8^ Intensive treatment was not associated with worse renal outcomes or greater adverse effects than standard treatment.^8^ There were no changes in diastolic function associated with adverse effects and this exploratory analysis has not changed the interpretation on safety.

## Discussion

This exploratory study presents trial data with findings regarding the influence of sustained lower BP on LV diastolic function in children with CKD. We performed a thorough assessment of diastolic function, encompassing mitral inflow, TDi on both lateral and septal segments, and LAVi. The results demonstrate that intensive BP control, has a positive impact on diastolic function, as evidenced by difference in the annual rate of change in E/A ratio, septal e', and LAVi between the intensive and the standard treatment arms.

Hypertension is one of the key determinants for cardiac remodelling in children with CKD.^16^, ^17^, ^18^ It is well established that adverse LV remodelling is associated with reduced diastolic function as a result of reduced LV compliance and elevated diastolic filling pressure.^19^^,^^20^ Data from the HOT-KID trial suggests that a target office systolic BP at the 50th percentile is close to the optimal target for preventing adverse cardiac remodelling as measured by RWT in children with CKD.^8^ The effects of intensive BP control on diastolic relaxation (E/A ratio), early septal diastolic motion (septal e’) and low diastolic filling pressure (LAVi) may be associated with improvement of LV remodelling as a result of lower BP. However, we found no correlation between change in LV structure and diastolic function (possibly because of limited power) and cannot determine whether these changes in diastolic function are driven by BP control or mediated by the effect of LV remodelling.

Diastolic function is most often measured by Spectral Doppler (E wave, A wave and E/A ratio) as measures of diastolic relaxation.^13^ We observed a reduction of E/A ratio in patients in the standard treatment arm, although none of the patients had E/A ratio <1, compared to unchanged E/A for those in the intensive treatment group over the course of the trial. Our results show that even small reductions in BP can affect diastolic function (even when within the normal range) and therefore BP control is likely to be critical in preventing diastolic dysfunction. The importance of our results lies in the context of a high proportion of children with CKD eventually developing clinically important diastolic dysfunction as they age and develop worsening renal function.^1^

Several observational studies have shown that elevated systolic BP is associated with a higher E/e’ ratio–another indicator of reduced LV diastolic function.^3^^,^^21^ However, in a recent study by Mitsnefes et al. of children with mild to moderate CKD and controlled hypertension, the E/e’ ratio was not statistically significantly associated with clinic BP.^1^ In our study, intensive BP compared to standard BP lowering did not reduce E/e’ (average, lateral and septal) ratios. These findings may reflect the already well-controlled BP at baseline with average BP close to the 50th percentile, associated with low prevalence of diastolic dysfunction with only 21·8% having E/average e’ > 8 and none E/average e’ >14 or E/A and e’/a’ ratios <1 at baseline.

Tissue Doppler imaging of myocardial diastolic motion (e’ and a’) is thought to be less dependent on loading conditions than some other techniques.^22^ Our finding of statistically significant difference in septal e’, but not in lateral e’ or average e’ between the two treatment groups is interesting and consistent with the data from the 4C study (Cardiovascular Comorbidity in Children with Chronic Kidney Disease) that the reduction of septal e’ compared to lateral e’ is more pronounced in children with CKD when compared to healthy children.^3^ The early diastolic relaxation in the septal segment may be more sensitive to haemodynamic or structural changes, while the lateral region remain unaffected due to differences in myocardial mechanics or loading conditions. Results from a recent systematic review found that septal e’ velocity was more strongly associated with increased Body Mass Index than lateral e’ or average e’.^23^ This reduction of early diastolic septal motion may be explained by preferential remodelling of the septum prior to the change in the lateral segment and needs further research.^24^

Previous studies have demonstrated that anaemia is associated with adverse cardiac remodelling and diastolic dysfunction possibly due to the reduction in oxygen-carrying capacity of the blood.^25^, ^26^, ^27^, ^28^ In our study, the haemoglobin level was similar between the two treatment groups at baseline, and there was no association of anaemia with diastolic function over the course of the trial. Therefore, it is unlikely that our results were explained by anaemia.

A key finding of our study is that a lower BP target resulted in a statistically significant reduction in LAVi in the intensive treatment arm compared to the standard arm. Elevated BP leads to increased afterload and subsequent LV remodelling. This remodelling process, characterised by changes in LV structure and diastolic dysfunction, is a well-documented consequence of hypertension. As hypertension persists, diastolic dysfunction is associated with impaired LV relaxation and increasing of LA pressure. Both Pulsed wave Doppler and TDi provide an acute assessment of diastolic function, whilst LAV reflects the duration and severity of increased LA pressure as an indicator of chronic diastolic dysfunction.^29^

Given its role as a surrogate measure of diastolic filling pressure, LAVi is one of the key criteria in the current diagnostic algorithm.^13^ In a small observational study, Daniels et al. reported that LA enlargement is prevalent in children with essential hypertension and is associated with higher systolic BP and increased LVM.^30^ Data from two large cohorts of children with CKD (HOT-KID and 4C) demonstrated that children with CKD stage 3 and 4 had significantly increased LAVi compared to healthy controls.^31^ Longitudinal observational studies in adults with CKD show that the increase of LA size predicts cardiovascular events.^32^^,^^33^ These findings collectively highlight the clinical relevance of LAVi in assessing diastolic function. By demonstrating a statistically significant reduction in LAVi when targeting a lower BP, we highlight the potential of intensified BP management in preserving cardiac health and preventing adverse outcomes associated with diastolic dysfunction.

The HOT-KID RCT was small and open-label, which may have affected the participant compliance, stress levels and perception of side-effects. We did not adjust for multiplicity and have interpreted this exploratory study using 95% confidence limits of the outcomes. There was a small amount of missing data; imputation analyses did not highlight any significant impact on our study findings, but we accept that we cannot exclude any bias from missing data. Although our analysis demonstrated a positive impact on diastolic function as a result of lower BP within an RCT, diastolic function measures were not defined as primary or secondary outcomes of the original HOT-KID trial. Therefore, our findings can only be considered as exploratory. The majority of our participants were of white race and a higher proportion of boys may limit the generalisation of our findings. The intensive group were treated with a greater number of anti-hypertensive drugs over the course of the trial, and we cannot distinguish a specific effect of BP reduction from a BP-independent effect of the antihypertensive drugs. The confidence intervals for change in lateral and average e’ were wide and we cannot therefore exclude a change in these measurements. We did not perform sub-sample analysis in children with different CKD aetiologies due to limited sample size. Haematology results were recorded annually as part of the trial, and we cannot exclude that participants may have had anaemia that was treated in the interim period.

In conclusion, our exploratory analysis of comprehensive diastolic function measures within the constraints of a small open label trial suggests that more intensive blood pressure control have a favourable impact on some measures of diastolic function in children with CKD. These subtle changes suggest that close monitoring of cardiac function may be useful in children with CKD, even in those with well-controlled BP according to current guidelines, to prevent progression into clinical diastolic dysfunction and heart failure. Further studies are needed to confirm our findings and to fully understand the long-term clinical implications of more intensive BP control.

## Contributors

MDS and PJC were the principal investigators, and JMS served as co-principal investigator. MDS and PJC designed the trial and study protocol. Together with HG, they collaborated with lead site investigators and local study teams to oversee site work, including recruitment, follow-up, and data collection. MDS, HG, and PJC supervised the study, contributed to data analysis, interpretation, and article revision.

HG, MDS, and PJC accessed, verified the data, and made the decision to submit the manuscript. The first draft was written by HG, MDS, and PJC. All authors reviewed and revised the manuscript and confirmed the accuracy and completeness of the data and adherence to the protocol. All authors read and approved the final version of the manuscript. The corresponding author had full data access and final responsibility for submission.

## Data sharing statement

The deidentified participant data that support the findings of this study can be made available upon reasonable request to the chief investigator (MDS).

## Declaration of interests

RDG received payments for lectures, expert testimony and support for attending meetings from Alexion Pharmaceuticals. RDG is on the Data safety monitoring board of Alexion Pharmaceuticals. RS received payments for lectures and support to attend meetings from Fresenius Medical care, Vitaflo and Amgen. RS received grants from Fresenious Medical Care and Vitaflo, and Royalties for Springer text book. RS is on the Fresenious Medical Care Scientific Advisory Board. PJC is the Vice President of British and Irish Hypertension Society. HG, JMS, JC, EF, AL, HM, HM, PS, JT, YT and MDS declare no conflicts of interest. No funding was received to support the HOT-KID study group.

## Appendix A Supplementary data

Supplementary Figure and Tables

Ebiomedicine_Protocol
