# Supplementary material for: An exploratory analysis on diastolic function in the intensive compared with less intensive blood pressure control to prevent adverse cardiac remodelling in children with chronic kidney disease (HOT-KID): a parallel-group, open-label, multicentre, randomised, controlled trial
Source: eBioMedicine. 2025 Apr 21;115:105691. doi: 10.1016/j.ebiom.2025.105691 (PMC12052684; doi:10.1016/j.ebiom.2025.105691)
Supplement: Supplementary Figure and Tables [file mmc1.docx]

**Supplement data**

**An exploratory analysis on diastolic function in the Intensive compared with less intensive blood pressure control to prevent adverse cardiac remodelling in children with chronic kidney disease (HOT-KID): a parallel-group, open-label, multicentre, randomised, controlled trial**

Haotian Gu^a*^, John M Simpson^a,b^, Janette Cansick^c^, Eric Finlay^d^, Rodney Gilbert^e^, Andrew Lunn^f^, Heather Maxwell^g^, Henry Morgan^h^, Mohan Shenoy^i^, Rukshana Shroff^j^, Pushpa Subramaniam^k^, Jane Tizard^l^, Yincent Tse^m^, Phil Chowienczyk^a^, Manish D Sinha^a,n*,^ on behalf of the HOT-KID study

**Affiliations**^:^

^a^King’s College London British Heart Foundation Centre, London, UK

^b^Department of Paediatric Cardiology, Evelina London Children’s Hospital, Guy’s & St Thomas
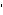
 NHS Foundation Trust, Westminster Bridge Road, London SE1 7EH

^c^Department of Paediatrics, Medway Maritime Hospital, Medway, UK

^d^Department of Paediatric Nephrology, Leeds General Infirmary, Leeds, UK

^e^Department of Paediatric Nephrology, Southampton General Hospital, Southampton, UK

^f^Department of Paediatric Nephrology, Nottingham University Hospital NHS Trust, Nottingham, UK

^g^Department of Paediatric Nephrology, Glasgow Royal Infirmary, Glasgow, UK

^h^Department of Paediatric Nephrology, Alder Hey Children's Hospital, Liverpool, UK

^i^Department of Paediatric Nephrology, Royal Manchester Children's Hospital, Manchester, UK

^j^Department of Paediatric Nephrology, UCL Great Ormond Street Hospital and Institute of Child Health, London, UK

^k^Department of Paediatrics, St Georges Hospital, Tooting, London, UK

^l^Department of Paediatric Nephrology, Bristol Royal Hospital for Children, Bristol, UK

^m^Department of Paediatric Nephrology, Great North Children’s Hospital, Newcastle Upon Tyne, UK.

^n^Department of Paediatric Nephrology, Evelina London Children’s Hospital, Guy’s & St Thomas
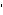
 NHS Foundation Trust, Westminster Bridge Road, London SE1 7EH.

*co-corresponding Authors

**Address for correspondence**:

Professor Manish D Sinha, King’s College London, Department of Paediatric Nephrology, 3^rd^ Floor Beckett House, Evelina London Children’s Hospital, Guy’s & St Thomas
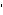
 NHS Foundation Trust, Westminster Bridge Road, LONDON SE1 7EH, United Kingdom.

Tel: +44 20 7188 4587

Email: [manish.sinha@nhs.net](mailto:manish.sinha@nhs.net)

Dr Haotian Gu, King’s College London, Clinical Pharmacology, 4^th^ Floor Northwing, St Thomas’ Hospital, Guy’s & St Thomas’ NHS Foundation Trust, London SE1 7EH, United Kingdom.

Tel: +44 20 71884799

Email: haotian.gu@kcl.ac.uk

**Funding**

British Heart Foundation (PG/11/90/28994) awarded to MDS; National Institution for Health and Care Research (NIHR303561) awarded to HG. The authors MDS, PJC acknowledge financial support from the Department of Health via the National Institute for Health Research (NIHR) comprehensive Biomedical Research Centre and Clinical Research Facilities awards to Guy’s and St Thomas’ NHS Foundation Trust in partnership with King’s College London and King’s College Hospital NHS Foundation Trust.

**Figure S1** CONSORT Diagram of the HOTKID study (1)

**
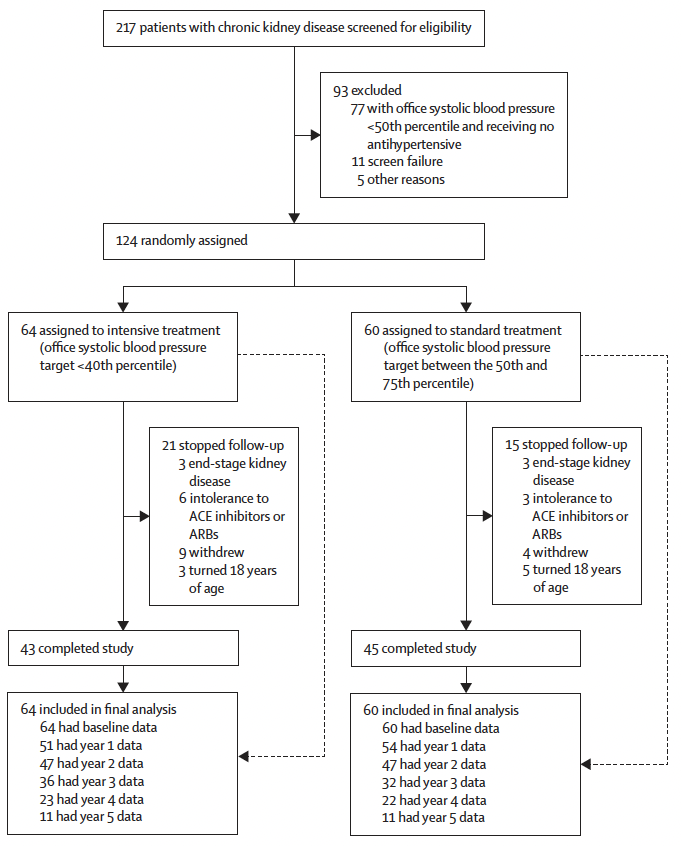
**

**Reference:**

1. Sinha MD, Gu H, Douiri A, Cansick J, Finlay E, Gilbert R, et al. Intensive compared with less intensive blood pressure control to prevent adverse cardiac remodelling in children with chronic kidney disease (HOT-KID): a parallel-group, open-label, multicentre, randomised, controlled trial. Lancet Child Adolesc Health. 2023;7(1):26-36.

**Table S1:** Diastolic function measures over time of the intention-to-treat population who completed 3-years of follow up

**a:** Change in mean E/A ratio from baseline.

| **E/A ratio** | **Intensive arm (SD)** | **Standard arm (SD)** | **Difference (CI)** |
| --- | --- | --- | --- |
| **Change in per year, mean** | 0·02 (0.04) | -0·05 (0·04) | -0·07 (-0·18, 0·03) |
| **Baseline (n=36, 32)** | 2·07 (0.57) | 1·95 (0.59) | -0·12 (-0·40, 0·16) |
| **Y1 (n=33, 32)** | 2·12 (0.63) | 1·93 (0.64) | -0·19 (-0·50, 0·13) |
| **Y2 (n=32, 28)** | 2·07 (0.51) | 1·94 (0.49) | -0·13 (-0·39, 0·13) |
| **Y3 (n=36, 32)** | 2·26 (0.61) | 1·89 (0.52) | -0·37 (-0·64, -0·11) |

**b:** Change in mean Septal e’ (m/s) from baseline.

| **Septal e’ in m/s** | **Intensive arm (SD)** | **Standard arm (SD)** | **Difference (CI)** |
| --- | --- | --- | --- |
| **Change in per year, mean** | 0.003 (0.001) | 0.0004 (0.001) | -0·003 (-0·001, 0·006) |
| **Baseline (n=36, 32)** | 0.125 (0.02) | 0.123 (0.01) | -0·002 (-0·01, 0·006) |
| **Y1 (n=33, 32)** | 0.134 (0.02) | 0.125 (0.02) | -0·008 (-0·02, 0·003) |
| **Y2 (n=33, 32)** | 0.139 (0.02) | 0.126 (0.02) | -0·013 (-0·02, -0·003) |
| **Y3 (n=36, 32)** | 0.134 (0.02) | 0.124 (0.02) | -0·01 (-0·02, -0·0004) |

| **Left atrial volume index (ml/m^2^)** | **Intensive arm (SD)** | **Standard arm (SD)** | **Difference (CI)** |
| --- | --- | --- | --- |
| **Change per year, mean** | -0.51 (0.34) | 0.25 (0.36) | 0·76 (-0·20, 1·72) |
| **Baseline (n=36, 32)** | 21.3 (5.49) | 19.5 (5.03) | -1.85 (-4·41, 0.70) |
| **Y1 (n=33, 32)** | 21.8 (6.15) | 18.0 (4.73) | -3.71 (-6.44, -0·99) |
| **Y2 (n=33, 32)** | 19.6 (5.30) | 20.8 (6.87) | 1.17 (-1·98. 4.32) |
| **Y3 (n=36, 32)** | 20.2 (7.65) | 19.4 (6.05) | -0·74 (-4·0, 2·52) |

**c:** Change in left atrial volume index (ml/m^2^) from baseline.

Means were estimated by use of a linear mixed effects model for repeated measures.

**Table S2.** Relationship between the annual rate of change in diastolic function and LV structure including baseline (and change) in LVMi or RWT.

|  | **Change in E/A** | | **Change in septal e'** | | **Change in LAVi** | |
| --- | --- | --- | --- | --- | --- | --- |
|  | **β** | **p** | **β** | **p** | **β** | **p** |
| **Age** | 0·082 | 0·322 | -0·076 | 0·374 | 0·026 | 0·763 |
| **Sex** | -0·117 | 0·147 | -0·76 | 0·357 | -0·024 | 0·774 |
| **Baseline LVMi** | 0·042 | 0·721 | -0·025 | 0·837 | 0·300 | 0·017 |
| **Baseline E/A** | -0·560 | <0·001 | - | - | - | - |
| **Baseline septal e’** | - | - | -0·551 | <0·001 | - | - |
| **Baseline LAVi** | - | - | - | - | -0·566 | <0·001 |
| **Change in LVMi** | -0·099 | 0·406 | 0·045 | 0·711 | 0·282 | 0·025 |

|  | **Change in E/A** | | **Change in septal e'** | | **Change in LAVi** | |
| --- | --- | --- | --- | --- | --- | --- |
|  | **β** | **p** | **β** | **p** | **β** | **p** |
| **Age** | 0·025 | 0·759 | -0·064 | 0·444 | -0·017 | 0·835 |
| **Sex** | -0·119 | 0·138 | -0·070 | 0·391 | 0·007 | 0·936 |
| **Baseline RWT** | 0·138 | 0·131 | -0·095 | 0·303 | -0·041 | 0·658 |
| **Baseline E/A** | -0·555 | <0·001 | - | - | - | - |
| **Baseline septal e’** | - | - | -0·541 | <0·001 | - | - |
| **Baseline LAVi** | - | - | - | - | -0·564 | <0·001 |
| **Change in RWT** | -0·063 | 0·491 | 0·074 | 0·427 | 0·220 | 0·020 |

LAVi: left atrial volum index; LVMi: left ventricular mass index; RWT: relative wall thickness.

**Table S3.** HOT-KID study group members.

| ***First names*** | ***Surnames*** |
| --- | --- |
| *Janette* | *Cansick* |
| *Abdel* | *Douiri* |
| *Eric* | *Finlay* |
| *Rodney* | *Gilbert* |
| *Haotian* | *Gu* |
| *Larissa* | *Kerecuk* |
| *Andrew* | *Lunn* |
| *Heather* | *Maxwell* |
| *Henry* | *Morgan* |
| *Reza* | *Razavi* |
| *Mohan* | *Shenoy* |
| *Rukshana* | *Shroff* |
| *Pushpa* | *Subramaniam* |
| *Jane* | *Tizard* |
| *Yincent* | *Tse* |
| *Poothirikovil* | *Venugopalan* |
| *John* | *Simpson* |
| *Phil* | *Chowienczyk* |
| *Manish* | *Sinha* |
