# Supplementary material for: An exploratory analysis on diastolic function in the intensive compared with less intensive blood pressure control to prevent adverse cardiac remodelling in children with chronic kidney disease (HOT-KID): a parallel-group, open-label, multicentre, randomised, controlled trial
Source: eBioMedicine. 2025 Apr 21;115:105691. doi: 10.1016/j.ebiom.2025.105691 (PMC12052684; doi:10.1016/j.ebiom.2025.105691)
Supplement: Ebiomedicine_Protocol [file mmc2.pdf]

Appendix

Submission type: Original contribution

Title: Impact of intensive blood pressure control on diastolic function in children with CKD in the HOT-KID trial

Short Title: Diastolic function in children with CKD

Authors: Haotian Gu[a*], John M Simpson[a,b], Janette Cansick[c], Eric Finlay[d], Rodney Gilbert[e], Andrew Lunn[f], Heather Maxwell[g], Henry Morgan[h], Mohan Shenoy[i], Rukshana Shroff[j], Pushpa Subramaniam[k], Jane Tizard[l], Yincent Tse[m], Phil Chowienczyk[a], Manish D Sinha[a,n*,] the HOT-KID investigators

Affiliations:

[a]King's College London British Heart Foundation Centre, London, UK

[b]Department of Paediatric Cardiology, Evelina London Children's Hospital, Guy's & St Thomas' NHS Foundation Trust, Westminster Bridge Road, London SE1 7EH

[c]Department of Paediatrics, Medway Maritime Hospital, Medway, UK

[d]Department of Paediatric Nephrology, Leeds General Infirmary, Leeds, UK

[e]Department of Paediatric Nephrology, Southampton General Hospital, Southampton, UK

[f]Department of Paediatric Nephrology, Nottingham University Hospital NHS Trust, Nottingham, UK

[g]Department of Paediatric Nephrology, Glasgow Royal Infirmary, Glasgow, UK

[h]Department of Paediatric Nephrology, Alder Hey Children's Hospital, Liverpool, UK

[i]Department of Paediatric Nephrology, Royal Manchester Children's Hospital, Manchester, UK

[j]Department of Paediatric Nephrology, UCL Great Ormond Street Hospital and Institute of Child Health, London, UK

[k]Department of Paediatrics, St Georges Hospital, Tooting, London, UK

[l]Department of Paediatric Nephrology, Bristol Royal Hospital for Children, Bristol, UK

[m]Department of Paediatric Nephrology, Great North Children’s Hospital, Newcastle Upon Tyne, UK.

[n]Department of Paediatric Nephrology, Evelina London Children’s Hospital, Guy’s & St Thomas' NHS Foundation Trust, Westminster Bridge Road, London SE1 7EH.

*co-corresponding Authors

Address for correspondence:

Professor Manish D Sinha, Kings College London, Department of Paediatric Nephrology, 3rd Floor Beckett House, Evelina London Children’s Hospital, Guy’s & St Thomas' NHS Foundation Trust, Westminster Bridge Road, LONDON SE1 7EH, United Kingdom.

Tel: +44 20 7188 4587

Email: manish.sinha@nhs.net

Dr Haotian Gu, King’s College London, Clinical Pharmacology, 4th Floor Northwing, St Thomas’ Hospital, Guy’s & St Thomas’ NHS Foundation Trust, London SE1 7EH, United Kingdom.

Tel: +44 20 71884799

Email: haotian.gu@kcl.ac.uk

Sources of Funding

British Heart Foundation (PG/11/90/28994); The authors acknowledge financial support from the Department of Health via the National Institute for Health Research (NIHR) comprehensive Biomedical Research Centre and Clinical Research Facilities awards to Guy's and St Thomas' NHS Foundation Trust in partnership with King's College London and King's College Hospital NHS Foundation Trust. There are no relationships with industry.

Conflicts of interest statement

We declare no competing interests. The authors have nothing to disclose.

Appendix 1: GCP Non CTIMP Protocol_HOTKID_v1.2 200714. This protocol is the protocol for the overall HOT-KID study including the HOT-KID trial.

Note to authors – this template is a guide – all the sections below must be included however this is not exhaustive, other sections may be added. Don't forget to delete the blue "guides" from each section! Once your protocol content is complete, update the index by clicking in the left of the field and pressing F9

PROTOCOL TITLE: **The Hypertension Optimal Treatment in Children with Chronic Kidney Disease study: The HOT-KID study- A randomised trial to compare effects of aggressive versus standard targets in blood pressure on target organ damage in children with CKD**

PROTOCOL IDENTIFICATION NUMBER (20/07/14 v1.2)

Sponsor

Name: Karen Ignatian, Guy's & St Thomas's NHS Foundation Hospitals NHS Trust
Address: R&D Department, Guy's Hospital, London SE1 7EH
Telephone:
Fax:
Email:: Karen.ignatian@gstt.nhs.uk

Chief Investigator

Name: Manish D Sinha
Address: Room 64, Sky Level, Department of Paediatric Nephrology, Evelina Children's Hospital, Westminster Bridge Road, London SE1 7EH
Telephone: 02071884587
Fax: 02071884591
Email: manish.sinha@gstt.nhs.uk

Name and address of Co-Investigator(s), Statistician, Laboratories etc

Name: Professor Phil Chowienczyk
Address: Department of Clinical Pharmacology, 4th Floor, North Wing, St Thomas's Hospital, London SE1 7EH
Telephone: 02071884771

Fax: 02071885116
Email: phil.chowienczyk@kcl.ac.uk

Name: Dr John Simpson
Address: 6th Floor Department of Paediatric Cardiology, Evelina Children's Hospital, Westminster Bridge Road, London SE1 7EH
Telephone: 02071887188
Fax:
Email: jmsimpsonmd@googlemail.com

Name: Professor Janet Peacock
Address: Division of Health and Social Care Research, Kings College London, 7th Floor, Capital House, 42 Weston Street, LONDON SE1 3QD UK
Telephone: +44 (0) 207 848 6651
Fax:
Email: janet.peacock@kcl.ac.uk

Name: Louise Watt
Address: Department of Clinical Pharmacology, 4th Floor, North Wing, St Thomas's Hospital, London SE1 7EH
Telephone: 02071884771
Fax: 02071885116
Email: louise.2.watt@kcl.ac.uk

Name: Professor Reza Razavi
Address: 4th Floor Lambeth Wing, St Thomas's Hospital, Westminster Bridge Road
London SE1 7EH
Telephone: +44 (0)20 7188 5440
Fax: +44 (0)20 7188 5442
Email: reza.razavi@kcl.ac.uk

CONTENTS

1. Background & Rationale.. **3**
2 Trial Objectives, Design and Statistics **4**
2.1. Trial Objectives.. **4**
2.2 Trial Design & Flowchart **4**
2.3 Trial Flowchart.. **4**
2.4 Trial Statistics **4**
2.4.1 Sample Size.. **5**
2.4.2 Randomisation **5**
2.4.3 Analysis.. **5**
3 Selection and Withdrawal of Subjects **5**
3.1 Inclusion Criteria **5**
3.2 Exclusion Criteria **5**
3.3 Withdrawal of Subjects... **6**
4 Assessment of Efficacy... **6**
4.1 Efficacy Parameters **6**
4.1.1 Primary Efficacy Parameters **6**
4.1.2 Secondary Efficacy Parameters **6**
4.2 Procedures for Assessing Efficacy Parameters.. **6**
5 Assessment of Safety **7**
5.1 Specification, Timing and Recording of Safety Parameters. **7**
5.2 Procedures for Reporting and Recording Adverse Events…………………………..7
6. Trial Steering Committee.. **7**
7. Direct Access to Source Data and Documents **7**
8. Ethics & Regulatory Approvals **7**
9. Quality Assurance, Data Handling, Publication Policy and Finance.......................... **8**
10. Signatures.. **8**

Study Synopsis

Title of clinical trial	The Hypertension Optimal Treatment in Children with Chronic Kidney Disease study: The HOT-KID study- A randomised trial to compare effects of aggressive versus standard targets in blood pressure on target organ damage in children with CKD.
Protocol Short Title/Acronym	The HOT-KID study
Study Phase if not mentioned in title	
Sponsor name	Guy's & St Thomas's NHS Foundation Trust
Chief Investigator	Dr Manish D Sinha
REC number	10/H0802/13
Medical condition or disease under investigation	Cardiovascular disease
Purpose of clinical trial	Interventional multi-centre UK-wide national trial<br>This study will determine whether controlling blood pressure to a lower level than is currently recommended will prevent the damage to arteries and heart.
Primary objective	1. a) Examine the relationship of LVM, cIMT and arterial stiffness to blood pressure (after adjustment for confounders) in a cohort of children with and without CKD.<br>b) Compare these measures in children with and without CKD when adjusted for peripheral and central blood pressure.<br>2. Perform a randomised controlled trial to determine whether aggressive blood pressure reduction (below the 40th percentile) compared to standard care (between the 50th-75th percentiles) is effective in normalising left ventricular mass, arterial structure/function.
Secondary objective (s)	Secondary objectives will be to examine the effects of aggressive versus standard blood pressure reduction on microalbuminuria and progression of renal failure and to determine if biomarkers of arterial injury/ventricular load are independently related to cardiovascular target organ damage.
Trial Design	Interventional multi-centre RCT UK-wide national trial
Endpoints	1. The primary endpoint of the RCT is to evaluate the benefit of aggressive blood pressure control on the normalization/ limitation of cardiovascular target organ damage as assessed by LV mass. The principal outcome

	measure will be the differences in LV mass between aggressive and standard treatment groups.<br>2. cIMT and PWV are secondary endpoints. Differences in cIMT and PWV will be secondary outcome measures.
Sample Size	150 (to give n=120 complete) and<br>n=150 age matched controls
Summary of eligibility criteria	Inclusion criteria:<br>aged 2 to 15 years with<br>CKD (stages 1-4 in the last 12 months)<br>+/- anti-hypertensive/s medications (irrespective of recent change/s in anti-hypertensive therapy) with none of the exclusion criteria outlined below.
Version and date of final protocol	Version 1 23/03/2012
Version and date of protocol amendments	Version 1.2 20/07/2014

1. Background & Rationale

This should comprise a brief description of the proposed study, a description of the population to be studied, the investigational product(s), device(s) or radiation exposure, a summary of findings from non-clinical studies that potentially have clinical significance, and from previous clinical trials that are relevant to the trial. A summary of the known and potential risks and benefits to human subjects should be presented, together with a justification for the choice of route of administration, dosage, dosage regimen, and treatment period(s). This should be supported by appropriate references to the published literature on the disease or condition, its treatment and the use of the study drug for the indication. Data from previous studies as well as any other information that provides background for the trial should be cited. A statement that the trial will be conducted in compliance with the principles of the Declaration of Helsinki (specifying which amendment), the principles of GCP and all (if any) applicable regulatory requirements is essential. It should also be stated that the protocol is to be submitted for approval by an NHS Research Ethics Committee (REC). The name of the Ethics Committee must be stated if the protocol has already been submitted to the Committee.

Chronic kidney disease (CKD) in children leading to end stage renal disease (ESRD) requiring dialysis and renal transplantation is one of the most important chronic diseases of childhood. At any time there are about 150 children with ESRD requiring regular dialysis in the United Kingdom, with 50-70 new patients commencing dialysis each year [1]. The number of children with CKD being treated in specialist paediatric nephrology units in the UK is not known but is probably 5-10 times more than the prevalent ESRD population. With modern management of chronic kidney disease and advancements in dialysis therapy, an increasing number of children are surviving through childhood and early adulthood. Thereafter, however, heart disease becomes one of the major causes of death in these young adults. The overall risk of cardiac death is about 700-times higher than in age-matched individuals from the normal population [2]. Cardiovascular disease (CVD) accounts for 40% of all deaths in this cohort and often presents as sudden cardiac arrest (not secondary to hyperkalemia or cerebrovascular accidents) [3-5]. Restoration of renal function by transplantation does not eliminate this increased risk and arterial disease in these adults is likely to relate to pre-clinical arterial disease, particularly arterial stiffening and left ventricular hypertrophy (LVH) developing during childhood.

In adult patients without CKD, predictors of CVD events are structural changes in the heart and arteries: LVH, increased intima-media thickening (IMT) and arterial stiffening that are strongly related to elevated blood pressure. These measures of target organ damage are similarly predictive in adults with CKD [6-8]. Arterial stiffness in particular is one of the most important predictors of CVD events. Blood pressure reduction in adults is highly effective at reducing the progression of CKD and CVD events but in later stages of CKD, it is effective only when accompanied by a reduction in stiffness of the arteries [7].

A high proportion of children with CKD already have evidence of target organ damage to heart and arteries. Mitsnefes *et al* [9] report an increase in the prevalence of LVH from 19% to 39% after 2-years of follow up. Small cross-sectional studies have reported increased carotid IMT in children with pre-dialysis CKD [12-16] and in young adults with childhood-onset ESRD [10,11] compared to controls without CKD. The most marked abnormalities have been observed in children with ESRD on dialysis [12,13,17], with longitudinal data in this group suggesting that there is improvement in cIMT post transplantation [16]. Increased arterial stiffness has been reported in children on dialysis [17,18] and in adults with childhood-onset ESRD [19]. In the adult patients stiffness was strongly related to blood pressure. However, other parameters thought to influence arterial stiffness such as parathormone levels, CRP, calcium containing phosphate binders and vitamin D [20-22] were not reported.

Despite a strong association between left ventricular mass, IMT and arterial stiffness with blood pressure in adults with and without CKD and in children without CKD [23], the role of blood pressure in determining target organ damage in children with CKD remains controversial. These are few data relating elevated blood pressure with IMT [16] and arterial stiffness in children with CKD. LVH and arterial stiffness are thought to be determined less by blood pressure and more by other mechanisms such as hyper-parathyroidism leading to myocardial hypertrophy and arterial stiffening through calcification [11-13,17,24].

The relationship of blood pressure with LVH in children with pre-dialysis CKD too remains unclear with the two largest multi-centre observational reports, one from Europe [24,25] and the other from the United States of America [26] showing conflicting relationships. The largest paediatric dataset is from the multi-centre North American 'CKiD study' that recently reported cross sectional data in 198 patients who had a single clinic BP measurement performed together with echocardiography and 24-hour ambulatory BP monitoring studies [26]. They observed LVH in 34% of the 36 subjects with confirmed hypertension (both clinic BP >95th percentile and ambulatory BP >95th percentile), 20% of 75 subjects with elevated ambulatory BP but normal clinic BP ("masked" hypertension) but only 8% of 83 subjects with normal clinic and ambulatory BP. They reported masked and confirmed hypertension to be significant independent predictors of LVH and concluded that casual BP alone was insufficient to predict the presence of LVH in children with CKD.

The multi-centre European trial, the 'ESCAPE' study aimed to evaluate the renoprotective efficacy of intensified blood pressure control in paediatric patients with pre-dialysis CKD. A significant reduction of renal failure progression was observed in patients who had intensified blood pressure control (<50th percentile) as opposed to conventional BP control (50th-95th percentile) with even better results in patients who achieved both a reduction in proteinuria and blood pressure to 'low-normal' 24-hour mean arterial pressure [25]. The ESCAPE study did not compare effects of aggressive versus standard BP control on LV mass or on arterial structure and function. However at baseline they found no association between LVH and BP [24,25]. In the ESCAPE study, LVH in children with CKD was associated with male gender, anaemia, ponderosity, hyperparathyroidism and reduced GFR but not hypertension [24] whilst the CKiD study reported hypertension defined by ambulatory BP monitoring as a significant risk factor [26]. These differences may be because of the difficulties in the measurement of blood pressure and the definition of "hypertension" during childhood as a categorical state with a value of systolic blood pressure above the 95th percentile for the population [23,27].

In our paediatric pre-dialysis CKD population at the Evelina Children's Hospital, we have recently reported on the significant association between LVH and blood pressure (**Figure 1, Appendix 1**) [28]. The mean LV mass index (LVMI) was 37.8 ± 9.1 g/m$^{2.7}$ in a cohort of 49 patients (29 male), aged 12.6y ± 3.0 (mean± sd), with eGFR 27.1± 12.2 ml/min/1.73m^2. Despite having clinic BP (CBP) measurements consistently within the currently accepted normal range (below 90th centile) there was a significant difference in BP (both clinic BP [**Figure 2, Appendix 1**] and 24-hour ambulatory BP monitoring) between those with and without LVH. 49% of our subjects were found to have LVH, as defined by recently published age specific LVMI ranges, related to systolic BP values measured both in clinic and by ambulatory BP monitoring. Patients with LVH had consistently higher BP values than those without ($p < 0.001$); although none were overtly hypertensive (>95th percentile). Thus, in our own study [28], we have found a strong correlation between systolic BP *within the normal range* and indexed LV mass (**Figure 1**). Similar to the CKiD study [26] we found high prevalence of LVH in subjects with masked hypertension but in contrast with the CKiD study, clinic systolic BP proved to be the strongest predictor of LVM; ambulatory BP did not add further predictive influence.

These data suggest that even in those with blood pressure below the 90th percentile, blood pressure may still be responsible for at least a component of LVH. The association of LVH with systolic BP in the absence of overt hypertension, suggests that current targets for BP control should be re-evaluated in this population. Extrapolation from data in adults with and without CKD and in children without CKD suggest that the same may be true for IMT and arterial stiffness.

Our findings are at variance with the ESCAPE study both with regard to the finding of a positive association of LVH with BP and lack of an association with male gender, anaemia, inflammation, ponderosity or hyperparathyroidism [28]. The strong correlation of blood pressure with LVH in our own population may in part be because all blood pressure measurements were performed by the same observer using auscultation with mercury sphygmomanometer or calibrated aneroid instruments. When using 24-hour ambulatory BP monitoring (ABPM) we (and the CKiD investigators) included all measurements as opposed to only a proportion of measurements (as in the ESCAPE study). We have previously shown that using a subset of the ABPM is likely to introduce significant clinical errors in the interpretation of ABPM [29].

Expert groups and international committees now recommend that, in adult patients with CKD lower blood pressure targets should be achieved than those aimed for general population [30,31]. These blood pressure targets are equivalent to the 50th-75th percentile in the general young adult population. In contra-distinction to practice in adults, until recently, recommendations for children with CKD were to maintain BP below the 90th percentile for the child's age, gender and height [32]. Based on the findings of the ESCAPE study, the European Society of Hypertension now recommend maintaining BP below the 75th percentile primarily to retard progression of renal failure [33]. Our findings of a high prevalence of LVH in children with CKD and BP below the 90th percentile, together with the experience in adults, raises the important question of whether even this target for children with CKD is too high. An additional consideration in children with CKD is the potential importance of central systolic blood pressure. Preliminary observations from ourselves suggest that central relative to peripheral systolic blood pressure is higher in children with CKD as compared to those without CKD. Because of the limitations of cross-sectional association studies, optimal hypertension control can only be answered by a randomised interventional trial. We propose testing the benefit of maintaining blood pressure below the 40th percentile on cardiovascular target organ damage by a randomised controlled trial.

EXPERIMENTAL DESIGN AND METHODS

Protocol

Subjects (n=150, to give n=120 complete) will be aged 2 to 15 years with CKD stages 1-4 in the last 12 months. Subjects will be eligible to enter the study whilst on anti-hypertensive/s medications irrespective of recent change/s in anti-hypertensive therapy. Detailed inclusion and exclusion criteria are described later in this document under 'Section 3'. Subjects with arterio-venous fistulae, on dialysis or those with a previous kidney transplant at the time of study entry will be excluded. eGFR will be

calculated using the Schwartz formula and CKD staged as per existing definitions [31,32]. Patients who reach end stage renal failure and commence dialysis or receive a kidney transplant will stop participating in the 'randomised controlled trial' but continue to have measurements performed until the end of the study. Informed consent/assent will be obtained from parents and patients (if age applicable) for participation in the study, as per institutional ethics committee approval. A control group (n=120) of age matched children without CKD will be included with all proposed study related investigations to be performed at baseline and at annual intervals. These will be recruited from siblings of study subjects and from a local community using links from a school based liaison project. In addition we aim to recruit a further control group (n=30) of subjects with systolic BP <50th percentile but on no anti-hypertensive medication.

All study related investigations at 'baseline' will be performed just before randomisation. Following enrolment and performance of baseline investigations children with CKD will be randomised to the two arms of the study: aggressive BP control (below the 40th percentile) or standard BP control (between 50th-75th percentile). A minimisation procedure will be used to ensure that patients randomised to the two arms are matched for: age, gender, CKD stage and proteinuria. Children will be recruited over the first 12-months of the study and will therefore have at least 2-years of follow up. Following randomisation all children in whom blood pressure is not to target will be reviewed at 2-4 weekly intervals. Thereafter all children will be monitored at least 4-monthly at the time of routine hospital visits and have clinic BP maintained in the assigned target range over the duration of the study.

Subjects recruited at other centres Subjects recruited in external centres will await 'research study visit' for performance of study related investigations and subsequent randomisation. Thus, any intervention as part of the research study will commence only following baseline investigations. All cardiac and vascular investigations described in this study will be performed by core group of study investigators at the ECH or at participating external centres.

On entry into the study a detailed history will be taken and case records reviewed to determine the cause and duration of CKD, past and family history relevant to CKD/CVD. Ethnicity (self defined by parents) will be recorded. The following measurements/records will be obtained on the first and subsequent visits:

(i) Clinical history, examination and anthropomometry
The clinical history will be updated together with details of current medications including hypertensive treatment, phosphate binders and hydroxylated vitamin D. A clinical examination will be performed. Height (stadiometre), weight, body mass index (BMI) and waist circumference will be recorded. Urine and blood biochemistry will be performed on the first and at annual visits.

(ii) Urinanalysis
Urine samples will be collected at study entry and at annual review. First morning voids on the three days prior to clinic visit will be required. The patient will keep urine specimens in a home refrigerator at 4 ºC before clinic visits. Specimens will be centrifuged to separate the cellular component from the supernatant and stored at -80 ºC. A mean value of three Ualb/Ucreat will be recorded.

(iii) Biochemistry
In addition to serum urea, electrolytes creatinine and cystatin C, the following biomarkers related to arterial injury, calcification and ventricular load will be determined:
a) Arterial injury: lipid profiles (total cholesterol, LDL-cholesterol, HDL-cholesterol, triglycerides and Apoliporotein-B), homocysteine, high sensitivity CRP.
b) Arterial calcification: plasma calcium, plasma phosphate and calcium-phosphate product (Ca*PO4), plasma intact parathyroid hormone (iPTH), FGF23, 25(OH) vit D, 1,25 (OH) vit D, blood haemoglobin (Hb).
c) Ventricular load: N-terminal pro-B type natriuretic peptide (NT-proBNP).

(iv) Clinic blood pressure and 24-hour ABPM

a) Clinic blood pressure will be taken as the mean of 3 measurements using the appropriate sized cuff and a calibrated aneroid instrument according to current guidelines. All observers will undergo appropriate training including the evaluation of their variation from independent experienced investigators. Normative criteria will be those defined in the Fourth report of the National High Blood Pressure Education Program Working Group in the United States ('Fourth report') [32] and will be expressed as z-scores.

b) 24-hour ABPM studies will be performed in patients > 5 years and > 120 cm using Spacelabs 90217 oscillometric ABP devices (Spacelabs Inc, Redmond, Wash, USA). The performance of 24-hour ABPM is not mandatory. Measurements will be performed once every 30-minute throughout the period of monitoring. Daytime and nighttime periods during each ABP recording will be defined using information in the patient 'ABPM diary card'. An ABPM study will be judged to be of poor quality and excluded if (1) if there are more than 3-hours of interrupted recordings at any time during the 24-hour period; (2) if the duration of ABPM recording is inadequate and less than 20-hours in total, less than 12-hours continuously or does not include any nighttime measurements. All mean ABPM parameters will be analysed as z-scores using the normative limits as per *Wuhl et al* [38]. Nocturnal systolic and diastolic dipping status will be defined as a reduction in nighttime systolic (or diastolic BP) of greater than 10% of the daytime systolic (or diastolic) BP. All ABPM studies will be analysed centrally at the ECH.

(v) Central blood pressure

Central blood pressure will be estimated from radial tonometry and modified BP measurement using Centron™ BP machine. We are currently performing a validation study to determine which method is most reliable.

(vi) Echocardiography

2D-guided M-mode echocardiography will be performed using images obtained in either parasternal long axis or short axis view of the left ventricle, as recommended by the American Society of Echocardiography [39]. ECHO studies will be performed by trained paediatric echocardiographers using a Philips iE33 ultrasound system (Philips Inc, Andover, Mass, USA). All studies will be stored digitally and analysed by a single investigator (JMS) who will be blinded to the medical history. Left ventricular mass will be calculated using the Devereux equation [40]. Left ventricular mass varies widely across the paediatric age range, therefore to allow standardisation it is usually expressed as left ventricular mass index (LVMI). We will use LVMI (LVM divided by height in meters raised to allometric power of 2.7 [$g/m^{2.7}$]) as a measure of LVH that accounts for body size [41]. LVH will be defined as LVMI ≥95th percentile using age-specific reference intervals for normal children [42], where appropriate, we will also calculate left ventricular mass for height z-scores [43]. Relative wall thickness (RWT) will be measured to assess the left ventricular geometry. Patients with increased LVMI (≥95th percentile) and elevated RWT (≥0.41), have concentric LVH; with increased LVMI (≥95th percentile) and normal RWT (<0.41) have eccentric LVH; and those with normal LVMI (<95th percentile) and elevated RWT (≥0.41) have concentric remodelling. Shortening fraction will be calculated to estimate the LV systolic function. Diastolic function will be assessed by pulsed Doppler interrogation of mitral valve inflow and tissue velocity imaging interrogation at the level of the mitral valve annulus both at the septum and left ventricular free wall [44]. The mitral valve E/e' ratio will be used as a surrogate for filling pressures. Cross sectional images in both four chambers and multiple short axis views will be obtained to permit analysis of myocardial rotation and torsion using two dimensional strain ("speckle tracking") techniques.

(vii) Carotid intima-media thickness

High resolution ultrasound (Linear transducer for iE33 (15-6MHz) will be used to obtain images of the common carotid artery. Mean common carotid intima-media thickness (cIMT) will be assessed using automated software (Medical Imaging Applications LLC, Iowa, USA) from digitized images obtained in diastole of the near and far walls of the both common carotid arteries 1-2 cm proximal to the flow divider [45,46]. This method is recognized as one of the most robust measures of IMT since the

reproducibility of common carotid IMT measurements is higher than that of other segments [47], and in most studies to date additional measurements from the bifurcation and internal carotid have not improved the predictive value of IMT [48,49].

(viii) Arterial stiffness
Arterial stiffness will be determined by measuring carotid-femoral pulse wave velocity (PWV) in children able to co-operate with this measurement and by measuring arm-thigh PWV in all subjects. Carotid-femoral PWV will be determined using the SphygmoCor system (Atcor medical, Australia) in which ECG referenced sequential carotid and femoral applanation tonometry is performed. This is usually well tolerated in children but some children are unable to keep still during carotid tonometry. An alternative technique (Vicorder, Skidmore Medical) employs simultaneous measurement of pulse waveforms from pressure cuffs placed around the arm and thigh. This method is particularly well tolerated in children. Although it is not an accepted standard we have shown that it is highly (correlation coefficient 0.7) correlated with carotid-femoral PWV obtained using the SphygmoCor system.

Blood pressure targets and drug therapy
Clinic BP measurements performed over a 3-month period immediately prior to entry to study will be used to calculate average Clinic BP percentile. All subjects participating in the study (with or without anti-hypertensive/s) will be randomly assigned to either 'aggressive' or 'standard' BP target study arms. All subjects will have clinic BP maintained in the assigned target range over the duration of the study. Up or down titration of blood pressure during the trial will be performed as per flowcharts in **Appendix 3**. This will be monitored at least 4-monthly at the time of routine hospital visits. Patients already on anti-hypertensive medications at the time of study entry will continue on these or change as per clinical indication. ACEi or ARB's will be the mandatory first line agents. The dose of the agent will be adjusted to achieve the target blood-pressure levels with initial follow up at 2-4 weekly intervals following commencement of medication. The following order of escalation will be used (1) calcium channel blocker (CCB) (2) beta-receptor blocker (3) others such as diuretic or an alpha channel blockers. The preferential use of long acting drugs with once daily dosing would be recommended. This order of escalation will be used unless there are clear clinical indications for other agents. Amendments to the 'BP titration' procedures (**Appendix 3**) will be made if necessary following review of data after 20 subjects.

REFERENCES

1. Lewis MA, Shaw J, Sinha MD, Adalat S, Hussain F, Castledine C, Schalkwyk DV, Inward C. UK Renal Registry 12th Annual Report (December 2009): Chapter 14 Demography of the UK Paediatric Renal Replacement Therapy population in 2008. Nephron Clin Pract 2010; 115 (suppl1):c279–c288. DOI: 10.1159/000301237.
2. Levey AS, Beto JA, Coronado BE, Eknoyan G, Foley RN, Kasiske BL, Klag MJ, Mailloux LU, Manske CL, Meyer KB, Parfrey PS, Pfeffer MA, Wenger NK, Wilson PW, Wright JT Jr. Controlling the epidemic of cardiovascular disease in chronic renal disease: what do we know? What do we need to learn? Where do we go from here? National Kidney Foundation Task Force on Cardiovascular Disease. *Am J Kidney Dis* 1998; 32(5):853-906.
3. McDonald SP and Craig JC. Long-term survival of children with end-stage renal disease. N Engl J Med 2004; 350: 2654-2662.
4. Groothoff JW, Gruppen MP, Offringa M et al. Mortality and causes of death of end-stage renal disease in children: a Dutch cohort study. Kidney Int 2002; 61: 621-9.
5. Parekh RS, Carroll CE, Wolfe RA and Port FK. Cardiovascular mortality in children and young adults with end-stage kidney disease. J Pediatr. 2002; 141: 191-7.
6. Blacher J, Guerin AP, Pannier B, Marchais SJ, Safar ME. Impact of aortic stiffness on survival in end-stage renal disease. *Circulation*. 1999;99:2434-2439.
7. Guerin AP, Blacher J, Pannier B, Marchais SJ, Safar ME, London GM. Impact of aortic stiffness attenuation on survival of patients in end-stage renal failure. *Circulation*. 2001;103:987-992.
8. London GM, Blacher J, Pannier B, Guerin AP, Marchais SJ, Safar ME. Arterial wave reflections and survival in end-stage renal failure. *Hypertension*. 2001;38:434-438.

9. Mitsnefes MM, Kimball TR, Kartal J, Witt SA, Glascock BJ, Khoury PR, Daniels SR. Progression of left ventricular hypertrophy in children with early chronic kidney disease: 2-year follow up study. J Pediatr 2006; 149:671-5.
10. Oh J, Wunsch R, Turzer M et al. Advanced coronary and carotid arteriopathy in young adults with childhood-onset chronic renal failure. Circulation 2002; 106: 100-105.
11. Briese S, Wiesner S, Will JC, Lembcke A, Opgen-Rhein B, Nissel R, Wernecke K-D, Andreae J, Haffner D and Querfeld U. Arterial and cardiac disease in young adults with childhood-onset end-stage renal disease- impact of calcium and vitamin D therapy. Nephrol Dial Transplant 2006; 21:1906-1914.
12. Litwin M, Wühl E, Jourdan C, et al. Altered morphologic properties of large arteries in children with chronic renal failure and after renal transplantation. J Am Soc Nephrol 2005; 16:1494-1500.
13. Mitsnefes MM, Kimball TR, Kartal J, et al: Cardiac and vascular adaptation in pediatric patients with chronic renal disease: Role of calcium-phosphorus metabolism. J Am Soc Nephrol 2005; 16:2799-2803.
14. Ziolkowska H, Brzewski M, Roszkowska-Blaim M. Determinants of the intima-media thickness in children and adolescents with chronic kidney disease. Pediatr Nephrol 2008; 23:805-811.
15. Muscheites J, Meyer AA, Drueckler E et al. Assessment of the cardiovascular system in pediatric chronic kidney disease: a pilot study. Pediatr Nephrol 2008;23:2233-2239.
16. Litwin M, Wühl E, Jourdan C, et al: Evolution of large-vessel arteriopathy in paediatric patients with chronic kidney disease. Nephrol Dial Transplant 2008; 23:2552-2557.
17. Shroff RC, Donald AE, Hiorns MP, et al. Mineral metabolism and vascular damage in children on dialysis. J Am Soc Nephrol 2007; 18: 2996-3003.
18. Covic A, Mardare N, Gusbeth-Tatomir P, Brumaru O, Gavrilovici C, Munteanu M, Prisada O and Goldsmith DJA. Increased arterial stiffness in children on haemodialysis. Nephrol Dial Transplant 2006; 21: 729-735.
19. Groothoff JW, Gruppen MP, Offringa M, De Groot E, Stok W, Bos WJ, Davin JC, Lilien MR, Van De Kar N, Wolff ED and Heymans HS. Increased arterial stiffness in young adults with end-stage renal disease since childhood. JASN 2002; 13: 2953-61.
20. Guerin AP, London GM, Marchais SJ, Metivier F. Arterial stiffening and vascular calcifications in end-stage renal disease. Nephrol Dial Transplant 2000; 15: 1014-1021.
21. London GM. Large arteries haemodynamics: conduit versus cushioning function. Blood Press 1997; Suppl 2:48-51.
22. Barenbrock M, Hausberg M, Kosch M, Kisters K, Hoeks AP, Rahn KH. Effect of hyperparathyroidism on arterial distensibility in renal transplant recipients. Kidney Int 1998; 54: 210-215.
23. Sinha MD, Reid CJD. Evaluation of blood pressure in children. Curr Opin in Nephrol Hypertens 2007; 16:577-584.
24. Matteucci MC, Wuhl E, Picca S, Mastrostefano A, Rinelli G, Romano C, Rizzoni G, et al; ESCAPE Trial Group. Left ventricular geometry in children with mild to moderate chronic renal insufficiency. J Am Soc Nephrol 2006; 17:218-26.
25. The ESCAPE trial group. Strict blood pressure control and progression of renal failure in children. N Engl J Med 2009; 361: 1639-50.
26. Mitsnefes M, FlynnJ, Cohn S, Samuels J, Blydt-Hansen T, Saland J, KimballT, Furth S, Warady B for the CKiD Study Group. Masked hypertension associates with left ventricular hypertrophy in children with CKD. *J Am Soc Nephrol* 2010; 21: 137-144.
27. Sinha MD, Reid CJD. At what level of blood pressure should hypertension be defined in children? Cardiol Young 2009; 29: 1-3.
28. Sinha MD, Tibby SM, Rasmussen P, Rawlins D, Turner C, Dalton N, Reid CJD, Rigden SPA, Booth CJB, Simpson JM. Blood pressure control and left ventricular mass in children with chronic kidney disease. Clin J Am Soc Nephrol 2011; 6: doi:10.2215/CJN.04690510
29. Jones H, Sinha MD. Misclassification of measurements during ABPM introduces significant errors in its interpretation. Pediatr Nephrol 2011; doi: 10.1007/s00467-011-1791-3.

30. Chobanian AV, Bakris GL, Black HR, Cushman WC, Green LA, Izzo JL Jr. Seventh Report of the Joint National Committee on Prevention, Detection, Evaluation, and Treatment of High Blood Pressure: The JNC7 Report. JAMA. 2003;289: 2560–2572.
31. 2003 European Society of Hypertension-European Society of Cardiology Guidelines for the management of arterial hypertension. J Hypertens 2003; 21:1011-53.
32. National High Blood Pressure Education Program Working Group on High Blood Pressure in Children and Adolescents. The Fourth Report on the Diagnosis, Evaluation and Treatment of High Blood Pressure in Children and Adolescents. Pediatrics 2004; 114:555–576.
33. Lurbe E, Cifkova R, Cruickshank JK, Dillon MJ, Ferreira I, Invitti C, Kuznetsova T, Laurent S, Mancia G, Morales-Olivas F, Rascher W, Redon J, Schaefer F, Seeman T, Stergiou G, Wühl E, Zanchetti A; European Society of Hypertension. Management of high blood pressure in children and adolescents: recommendations of the European Society of Hypertension. J Hypertens 2009; 27(9):1719 -1742.
34. Vickery S, Stevens PE, Dalton RN, van Lente F, Lamb EJ. Does the ID-MS traceable MDRD equation work and is it suitable for use with compensated Jaffe and enzymatic creatinine assays? *Nephrol Dial Transplant* 2006; 21: 2439-2445.
35. Schwartz GJ, Haycock GB, Edelmann CM Jr, Spitzer A. A simple estimate of glomerular filtration rate in children derived from body length and plasma creatinine. Pediatrics 1976; 58(2):259-63.
36. Booth C, Turner C, Dalton RN. Estimated glomerular filtration rate (eGFR) in children. Pediatr Nephrol 2007; 22(9): 1543: 534(P) [Abstract].
37. Hogg RJ, Furth S, Lemley KV. National kidney foundation’s kidney disease outcomes quality initiative clinical practice guidelines for chronic kidney disease in children and adolescents: evaluation, classification, and stratification. Pediatrics 2003; 11: 1416–1421.
38. Wuhl E, Witte K, Soergel M, Mehls O, Schaefer F, Kirschstein M, Busch C, Danne T, Gellermann J, Holl R, Krull F, Reichert H, Reusz GS, Rascher W; German Working Group on Pediatric Hypertension. Distribution of 24-h ambulatory blood pressure in children: normalised reference values and role of body dimensions. J Hypertens. 2002; 20: 1995-2007.
39. Lang RM, Bierig M, Devereux RB, Flachskampf FA, Foster E, Pellikka PA, Picard MH, Roman MJ, Seward J, Shanewise JS, Solomon SD, Spencer KT, Sutton MS, Stewart WJ (2005) Recommendations for chamber quantification: a report from the American Society of Echocardiography’s Guidelines and Standards Committee and the Chamber Qualification Writing Group, developed in conjunction with the European Association of Echocardiography, a branch of the European Society of Cardiology. J Am Soc Echocardiogr 18: 1440-1463.
40. Devereux RB, Alonso DR, Lutas EM, Gottlieb GJ, Campo E, Sachs I, Reichek N. Echocardiographic assessment of left ventricular hypertrophy: comparison to necropsy findings. Am J Cardiol 1986;57: 450–458.
41. de Simone G, Daniels SR, Devereux RB, Meyer RA, Roman MJ, de Divitiis O, Alderman MH. Left ventricular mass and body size in normotensive children and adults: assessment of allometric relations and impact of overweight. J Am Coll Cardiol 1992;20: 1251–1260.
42. Khoury PR, Misnefes M, Daniels SR, Kimball TR. Age-specific reference intervals for indexed left ventricular mass in children. *J Am Soc Echocardiogr* 2009; 22(6): 709-14.
43. Foster BJ, Mackie AS, Mitsnefes M, Ali H, Mamber S, Colan SD. A novel method of expressing left ventricular mass relative to body size in children. *Circulation* 2008; 117(21):2769-75.
44. Eidem BW, McMahon CJ, Cohen RR, Wu J, Finkelshteyn I, Kovalchin JP, Ayres NA, Bezold LI, Smith EO and Pignatelli RH. Impact of cardiac growth on Doppler tissue imaging velocities: a study in healthy children. J Am Soc Echo-Cardiogr 2004; 17:212-21.
45. Mancini GB, Dahlof B, Diez J. Surrogate markers for cardiovascular disease: structural markers. *Circulation*. 2004;109:IV22-IV30.
46. Bots ML, Hoes AW, Koudstaal PJ, Hofman A, Grobbee DE. Common carotid intima-media thickness and risk of stroke and myocardial infarction: the Rotterdam Study. *Circulation*. 1997;96:1432-1437.
47. O'Leary DH, Polak JF, Kronmal RA, Manolio TA, Burke GL, Wolfson SK, Jr. Carotid-artery intima and media thickness as a risk factor for myocardial infarction and stroke in older adults. Cardiovascular Health Study Collaborative Research Group. *N Engl J Med*. 1999;340:14-22.

48. Del Sol AI, Moons KG, Hollander M, Hofman A, Koudstaal PJ, Grobbee DE, Breteler MM, Witteman JC, Bots ML. Is carotid intima-media thickness useful in cardiovascular disease risk assessment? The Rotterdam Study. *Stroke*. 2001;32:1532-1538.
49. Stork S, van den Beld AW, von Schacky C, Angermann CE, Lamberts SW, Grobbee DE, Bots ML. Carotid artery plaque burden, stiffness, and mortality risk in elderly men: a prospective, population-based cohort study. *Circulation*. 2004;110:344-348.
50. Fagard RH, Celis H, Thijs L, Wouters S. Regression of Left Ventricular Mass by Antihypertensive Treatment. A Meta-Analysis of Randomized Comparative Studies. *Hypertension* 2009; 54:1084.

Also see Figures in Appendix 1: 26/01/2012 version 1

2 Trial Objectives, Design and Statistics

2.1. Trial Objectives

This should comprise specific statements of the purpose (ie aims and objectives) of the study, together with a definition of the primary (and secondary) endpoints of the study.

(See also 4.1.1 and 4.1.2 of this protocol)

Primary objectives

1. a) Examine the relationship of LVM, IMT and arterial stiffness to blood pressure (following adjustment for confounders) in a cohort of children with and without CKD.
   b) Compare these measures in children with and without CKD when adjusted for peripheral and central blood pressure.

2. Perform a randomised controlled trial to determine whether aggressive blood pressure reduction (below the 40th percentile) compared to standard care (below the 75th percentile) is effective in normalising left ventricular mass, arterial structure/function.

Secondary objectives will be to examine the effects of aggressive versus standard blood pressure reduction on microalbuminuria and progression of renal failure and to determine if biomarkers of arterial injury/ventricular load are independently related to cardiovascular target organ damage.

2.2 Trial Design & Flowchart (Appendix 2)

A description of the design of trial to be conducted (eg double-blind, single-blind, open label, placebo-controlled, parallel-group, double-dummy, cross-over, etc.) should be given. Please also include a schematic diagram (flow chart) of trial procedures and stages is desirable as it is particularly useful for determining activities involved during each clinic visit (eg blood tests or scans, treatment, diary completion, adverse event monitoring, physical examination etc).

The principal outcome measure for the controlled trial will be the differences in LV mass between aggressive and standard treatment groups.

Differences in IMT and PWV will be secondary outcome measures.

2.3 Trial Flowchart

Please include a time/event matrix (flow chart) of trial procedures and stages. This desirable as it is particularly useful for determining activities involved during each clinic visit (eg blood tests or scans, treatment, diary completion, adverse event monitoring, physical examination etc).

<u>Please see Appendix 2 26/01/2012 version 1 with this document</u>

Key task completion date (months)

	Day 1	4-mths	8-mth	1 year	2 year	3 year
Patient information and informed consent	Over first 12 months of study					
Physical examination	At recruitment and at least 4 monthly					
Demographics	√	√	√	√	√	√
Clinic BP and review of medications	√			√	√	√
ABPM (in sub-group)	√			√	√	√
Central BP evaluation	√			√	√	√
Blood sample	√			√	√	√
Urine sample	√			√	√	√
Non-invasive arterial studies (cIMT and PWV)	√			√	√	√
Echocardiography	√			√	√	√
24-hour ambulatory BP monitoring	√			√	√	√

All subjects will have clinic BP maintained in the assigned target range over the duration of the study. Up or down titration of blood pressure during the trial will be performed as per flowcharts in **Appendix 3**. Amendments to the 'BP titration' procedures (appendix 3) will be made if necessary following review of data after 20 subjects.

2.4 Trial Statistics

A description of the measures taken to avoid, or at least minimize, bias should be given. Specifically, this normally means describing the procedure(s) for randomisation and blinding. A description of the statistical methods to be employed, including timing of any planned interim analyses should also be provided. The number of subjects to be enrolled (in multicentre trials, the numbers of subjects for each site) should be stated, together with the rationale for the sample size (the "power calculation"). The level of significance that is to be used in each trial analysis must be stipulated, together with the procedure(s) for accounting for any missing, unused, and spurious data. Procedures for reporting any deviation from the original statistical plan should be described and justified. The data set for any analysis must be clearly stipulated (eg "all subjects", "randomised subjects", "intent to treat") and the population(s) should be clearly defined.

Differences in outcome will be estimated using a random effects (mixed) model that includes the baseline value of the outcome. Sensitivity analyses will be performed to also include key prognostic variables including known factors other than blood pressure and those identified by univariate analyses.

2.4.1 Sample Size

The outcome data are longitudinal and so we will fit a model that takes account of this data structure. The random effects model is a 2-level multilevel model that allows for the repeated data within subjects, while allowing some measurements to be missing. (This is preferred to repeated measures analysis of variance that requires complete data on all subjects). For a particular outcome, eg LVmass, the baseline value for each subject will be included in the model to adjust for (random) baseline variability between subjects. The results of the analysis are in the form of mean difference in LVmass between the intervention and control groups, at the endpoint, with a 95% CI.

The original sample size calculations were based upon longitudinal measurements of LVMI at annual intervals in our cohort of children with CKD (n=47). The SD of change in LVMI is 7.7 g/m$^{2.7}$ and this was obtained without the same standardized reading of LVM that we are proposed in the study. To be conservative we assume just a 20% improvement on our existing data giving an SD of 6 g/m$^{2.7}$ result from our own cohort. Thus with n=60 in each group we should be able to

detect a difference in change in LVMI of > 3.1 g/$m^{2.7}$ (=9% of baseline LVMI) over the 2 year duration with 90% power (P<0.05). This is one third of the difference (9.4 g/$m^{2.7}$) between subjects in our cohort that have blood pressure in the 50-75th and below 40th percentiles. Although we are assuming relatively rapid tracking of LV mass in relation to blood pressure, this is not unreasonable given the time frame of LV regression in adults. A recent meta-analysis [Fagard et al, *Hypertension* 2009; 54:1084] of randomized studies shows a mean change in LVMI of 10.3% over duration of 6 months.

Interim analysis to be performed at 1-year by the statistical team to check if the SD of measured indexed LV mass (LVMI) is in keeping with initial projections. The choice of standard deviation (SD) for the initial sample size was made using the best data available at the time of the study design. In accordance with good practice, we will monitor the SD during the study across the whole study group i.e. NOT by randomised group. In this way we can verify that the overall SD observed in the trial is in fact as expected. If the SD is found to be very different, either larger or smaller, we will consider whether the sample size calculations need to be revised accordingly. In this way we will be able to be as certain as is possible that the trial is powered as planned and ensure that the primary study endpoint is achieved We will inform the Research Ethics Committee of the results of the interim analysis soon after the end of the 1st year.

With regard to PWV, the within subject SD for measurements separated by several months is 0.5 m/s so we should be able to detect a difference in change in PWV of 0.3 m/s. Although we do not have data relating PWV to level of blood pressure, this difference is small in absolute terms (being equivalent to only a few years of “vascular ageing” in adults) and therefore we feel it is likely that we will be able to detect a meaningful change in PWV.

The following research team members will be ‘blinded’ to the blood pressure percentile of the patient: (i) technologist/s performing the cardiac and vascular measurements and (ii) Research team member entering data to MedSciNet. The clinical team will also be kept blinded to the results of the echocardiogram and vascular studies.

2.4.2 Randomisation

Randomisation will be performed using the Institute of Psychiatry CTU online platform. Following consent baseline measurements will be performed immediately *before* randomisation At external centres visiting investigators will perform measurements immediately before randomisation.

2.4.3 Analysis

As outlined above.

(i) Appointment of ‘Data Monitoring Committee’ (DMC). The members of this committee will include independent observers as per routine guidelines. The DMC will review the results of the proposed interim analysis at 1-year following commencement of study.

(ii) Interim analysis will also review data regarding the safety of proposed study arms at 1-year. The results will be reviewed by DMC and used to inform REC and the wider research team.

(iii) Both SPSSv17 and SigmaStat v10 will be used for statistical analysis, in close consultation with statisticians at Kings College London.

3 Selection and Withdrawal of Subjects

3.1 Inclusion Criteria

This section should contain details of age, sex, disease, prior treatment constraints etc., under which a subject is deemed to be suitable (eligible) to participate in the trial. This also includes healthy volunteers and any “control”

groups etc. Each such "group" should be defined separately. Informed consent to participate (preferably written and witnessed) must be stated as an inclusion criterion. A simple list format is the preferred style.

i. aged 2 to 15 years with
ii. chronic kidney disease with stages 1-4 the last 12 months and
iii. with or without anti-hypertensive/s medications (irrespective of recent change/s in anti-hypertensive therapy). Subjects on anti-hypertensive medications must be able to tolerate either an ACEi or ARB's.
iv. subjects with average clinic systolic BP <50th percentile and on no antihypertensive medication will be eligible as 'CONTROL' subjects only
v. other 'CONTROL' subjects will include children with normal renal function and blood pressure attending hospital for unrelated medical review or siblings of subjects with CKD

3.2 Exclusion Criteria

This section should contain details of age, sex, disease, prior treatment constraints etc., under which a subject is considered to be unsuitable for inclusion into the study population. (Examples frequently include exposure to prior radiotherapy in cancer trials, and participation in a trial with an investigational product within the previous three months in pharmacokinetics trials). A simple list format is the preferred style.

i. age <2 and >15 years
ii. subjects who have/had an arterio-venous fistulae,
iii. subjects who have/had are on dialysis
iv. subjects who have/had a functioning kidney transplant
v. patients with symptomatic BP or with past history of difficulty to control BP or
vi. patients in whom there is a clinical urgency to treat BP and inclusion in study may result in possible delay of treatment
vii. patients with arrhythmia or clinical heart failure
viii. patients with known structural cardiac abnormality
ix. subjects on treatment with angiotensin converting enzyme inhibitor (ACEi) or angiotensin receptor blocker (ARB) agents for treatment of proteinuria only or
x. subjects who are likely to be of clinical concern following up or down titration of BP levels as described in 'Appendix 3'
xi. subjects who are unable or intolerant to performance of study measurements e.g. height, echo or PWV
xii. subjects who have/had intolerance to Angiotensin converting enzyme inhibitors (ACEi) and ARB's drug/s or have any existing contraindications

3.3 Withdrawal of Subjects

Please provide details of when and how to withdraw subjects from the trial or investigational product treatment. It is essential that you specify the type and timing of the data to be collected for withdrawn subjects and indicate whether (and if so, how) withdrawn subjects are to be replaced, as well as arrangements for safety assessment follow-up of any subjects withdrawn from the trial as a result of (Serious) Adverse Events.

i. Subjects will be withdrawn if they are unable to tolerate the performance of study related measurements e.g. height, echo or PWV or if they develop concerning adverse effects as a result of ACEi/ ARB's class of anti-hypertensive drug/s used as part of taking part in the study.
ii. If a subject wishes to withdraw from the study this will be allowed. Identifiable data already collected with consent would be retained and used in the study. No further data or tissue would be collected or any other research procedures carried out on or in relation to the participant.

iii. In principle we would aim to keep blood and urine specimen and data gathered up to the point that the consent is lost. However as the patients are continually followed up by the clinical team we will be able to determine if the patient wants us to withdraw their data from the study. We will always follow the patient and/or their families wishes.

4 Assessment of Efficacy

4.1 Efficacy Parameters

Describe the measures that will be used to determine the efficacy of treatment (eg glucose, blood pressure, tumour reduction etc). Primary efficacy parameters should be stated first, then any secondary parameters and "surrogate markers" listed in 4.1.2.

4.1.1 Primary Efficacy Parameters

N/A

4.1.2 Secondary Efficacy Parameters

N/A

4.2 Procedures for Assessing Efficacy Parameters

Describe here the procedures for determining the primary (and secondary) efficacy parameters (eg venepuncture, 25 mls to be drawn 12 hrs post treatment for full blood count, SMAC and glucose, or CT scan at 6 months, etc). State also here what is to be recorded in the CRF (eg WBC, Hb, Urea, Glucose etc rather than "FBC" or "SMAC") – ie stipulate the parameters that are actually to be used for the analysis of efficacy. (See also Safety Parameters, 5.1 below)

N/A

5 Assessment of Safety

5.1 Specification, Timing and Recording of Safety Parameters.

Describe the measures that will be used to determine subject safety during the study. These will include physical examination, blood tests and adverse event reporting. Obviously, there will be close correlation with efficacy testing intervals and efficacy blood tests etc. In this section the tests that to be performed for assessing the safety of the subject should be appropriate to the treatment (eg WBC and platelets in chemotherapy, LFTs if there is a known or suspected risk of hepatotoxicity, U&Es if there is a risk of renal problems etc), although "general" assessments from FBC or SMAC would be acceptable. As with efficacy measures, please stipulate (ie repeat even if given above) the times at which safety evaluations will be conducted (Please do not write "see above" in this section; please copy & paste from 4.2 as and where appropriate).

All subjects will have clinic BP maintained in the assigned target range over the duration of the study. Up or down titration of blood pressure during the trial will be performed as per flowcharts in **Appendix 3**. Amendments to the ‘BP titration’ procedures (appendix 3) will be made if necessary following review of data after 20 subjects.

Adverse events will be assessed at each study visit.

Important expected adverse events will be actively surveyed (i.e. must be assessed to complete the case report form).

An independent Data Monitoring Committee (DMC) will review adverse event data annually or more frequently if requested by the DMC.

5.2 Procedures for Recording and Reporting Adverse Events

Investigators will assess the seriousness and causality (relatedness) of all AEs experienced by the patient. Definitions of different types of AE are listed below.

Adverse Events (AEs): An adverse event is any untoward medical occurrence in a subject as a result of taking part in the research study (study medication, application of the study device) or within a previously specified period of time after the treatment has been completed. An AE can therefore be any unfavourable and unintended sign (including an abnormal laboratory finding), symptom or disease temporally associated with the use of medication as part of taking part in the study. As the safety profiles of the anti-hypertensive agent/s used in this trial are well described, only Adverse Reactions (ARs) experienced during treatment will be reported.
Pre-existing condition/s, diagnostic and therapeutic procedures, such as surgery (although the medical condition for which the procedure was performed must be reported if new) performed in the routine management of the subjects condition are not defined as AE. Any worsening of any pre-existing condition/s will be reported.

Adverse Reactions (ARs) An AR is an AE if judged as having a reasonable causal relationship to the anti-hypertensive agent/s. This usually means that there is evidence or argument to suggest a causal relationship.

Serious Adverse Events (SAEs): An SAE is any AE that: (i) Results in death; (ii) Is life threatening (an event in which the patient was at risk of death at the time of event); (iii) Requires in-patient hospitalisation or prolongation of existing hospitalization; (iv) Results in persistent or significant disability or incapacity; (v) Results in a congenital anomaly or a birth defect (vi) Or is otherwise considered medically significant by the Investigator.

Important AEs that are not immediately life threatening or do not result in death or hospitalisation but may harm the subject or may require intervention to prevent one of the other outcomes listed above, should be considered serious.

The following SAEs do not require immediate reporting by the site and are not regarded as unexpected for the purpose of this trial. Events <u>not</u> considered SAEs are any period of hospitalizations for (i) Routine treatment/ monitoring of the condition, not associated with any deterioration in condition and (ii) Treatment, which was elective or pre-planned, for a pre-existing condition that is unrelated to the indication under study, and has not worsened.

Expected SAEs are those listed in the current IB of the used anti-hypertensive agent/s. These are available at http://emc.medicines.org.uk/. Further information about anti-hypertensive agents can also be obtained at http://www.bnf.org/bnf/index.htm

Serious Adverse Reactions (SARs): A SAR is an SAE if a reasonable causal relationship can be made to the anti-hypertensive agent. This usually means that there is evidence or argument to suggest a causal relationship. Factors to consider when assessing causality of SARs include the nature and timing of the reaction and its relationship to the dose of the anti-hypertensive medication.

Suspected Unexpected Serious Adverse Reactions (SUSARs)

A SUSAR is an SAR, which is of a type or severity which is not consistent with the up-to-date product information in IB.

Details of all other SAEs will be documented and reported from the date of commencement of protocol defined treatment until three months after the administration of the last treatment.

Assessing Severity and Causality of AEs and SAEs

All AEs and SAEs should be evaluated by a doctor to determine severity and causality between the anti-hypertensive agent/s and/or concomitant therapy and the AE.

Reporting all Types of Adverse Events

1. **Reporting Adverse Events/Reactions**

Adverse events relating to the patients underlying disease and its treatment will be assessed at each study visit, and recorded on the follow-up data collection form. The study investigators will provide details of all adverse events to the Data Monitoring Committee (DMC) for their review on an annual basis.

2. **Reporting Serious Adverse Events/Reactions**

All SAEs must be recorded on the adverse event form and faxed to the CI within 24 hours of the research staff becoming aware of the event. Please ensure that the local Principal Investigator has assigned causality and expectedness to the SAE before reporting. For each SAE, the following information will be collected: (i) Full details in medical terms with a diagnosis, if possible. (ii) Action taken (iii) Outcome (iv) Causality, in the opinion of the investigator* and (v) Whether the event would be considered expected or unexpected*

*Assessment of causality and expectedness **must be made by a doctor**. If a doctor is unavailable, initial reports without causality and expectedness assessment should be submitted to the CI by a healthcare professional within 24 hours, but must be followed up by medical assessment as soon as possible thereafter.

Reporting SUSARs

SAEs classed by the local investigator as both suspected to be related to the anti-hypertensive agent/s and unexpected are SUSARs, and are subject to expedited reporting. The investigator should complete a SAE form, and fax to the CI within 24 hours of the research staff becoming aware of the event.

The Chief Investigator (or nominated individual) will undertake urgent review of SUSARs within 24 hours of the event being reported. The Chief Investigator will not overrule the causality, expectedness or seriousness assessment given by the local investigator but may comment on these and can upgrade if deemed appropriate.

Data and Safety Monitoring Plan

An independent Data Monitoring Committee (DMC) will be established for this trial and will include members as per standard guidelines and include at least one member from each of the following

designations: a statistician, an expert in renal disease and an expert in cardiovascular medicine. The DMC will meet annually to review all collected data and may meet more frequently if required after analysis of the available data. The DMC will advise the Trial Management Committee and the independent Trial Steering Committee on the safety of continuing this clinical trial.

6. Trial Steering Committee

Define function of committee and list names etc

This committee will comprise of CI (Dr Sinha), at-least two of other co-applicants (Drs Simpson, Goldsmith and Professor Chowienczyk), research nurse (TBA), research assistant/s (Laura Milne, Louise Watt and TBA) and trial statistician (Professor Peacock). The committee will meet regularly to monitor recruitment and completion of study objectives with formal meetings annually to monitor the data quality, safety and achievement of study objectives.

7. Direct Access to Source Data and Documents

The sponsor should ensure that it is specified in the protocol (or reference is made to another written agreement) that the investigator(s) and the institution(s) will permit trial-related monitoring, audits, REC review, and regulatory inspections (where appropriate) by providing direct access to source data and other documents (ie patients' case sheets, blood test reports, X-ray reports, histology reports etc).

8. Ethics & Regulatory Approvals

A statement that the trial will be conducted in compliance with the principles of the Declaration of Helsinki (specifying which amendment), the principles of GCP and any applicable regulatory requirements (specify current legislation) is essential. State the name and address of the REC to which the study protocol and other documentation will be submitted (eg Liverpool Adult Research Ethics Committee or Liverpool Children's Research Ethics Committee). You should also state that any subsequent protocol amendments will submitted to the REC, and that you provide the REC with progress reports, and a copy of the Final Study Report

This clinical trial will be conducted in accordance with the ethical principles as per the Declaration of Helsinki, and the principles of Good Clinical Practice and all applicable requirements as stated in the R&D approval. The Ethics for this study have been approved by the NRES Committee London – Westminster (REC reference 10/H0802/13).
Any subsequent protocol amendments will be submitted to the REC and progress reports and copy of final study report will be provided to REC as per standard requirements.

9. Quality Assurance, Data Handling, Publication Policy and Finance

Give details as to how QC will be maintained, data handling procedures, publication policy and finance.

Analysis:

(i) Professor Janet Peacock, Professor of Medical Statistics, King's College London and her team will provide high level statistical input to the trial, overseeing the randomisation and formulating the analysis plan. Differences in outcome will be compared by a random effects (mixed) model. Professor Peacock will formulate a detailed analysis plan during the work-up phase of the study.

(ii) Interim analysis to be performed at 1-year by statistical team to check if SD of measured indexed LV mass (LVMI) is in keeping with initial projections.

(iii) *Appointment of 'Drug monitoring committee' (DMC)*. The members of this committee will include independent observers as per routine standards. The DMC will review the results of the proposed interim analysis at 1-year following commencement of study.

(iv) Interim analysis will also review data regarding the safety of proposed study arms at 1-year. The results will be reviewed by DMC and used to inform REC and the wider research team.

Data handling:

(i) Data will be entered on an online data entry platform 'MedSciNet'. This will be overseen by Mr Bola Coker, at the Biomedical Research Centre at King's Health Partners and Professor Janet Peacock. Mr Coker will also help with the safe running of study database and collation of results.

(ii) The following research team members will be 'blinded' to the blood pressure percentile of the patient.
   a. All technologist/s performing the cardiac and vascular measurements
   b. Research team member entering data to MedSciNet

(iii) The clinical team will also be kept blinded to the results of the echocardiogram and vascular studies.

10. Signatures

To be signed by Chief Investigator minimum and statistician if applicable.

______________________________ ____________________

Chief Investigator Date

Print name

______________________________ ____________________

Statistician Date

Print name
